# Quantitative proteomic analysis reveals the ethanologenic metabolism regulation of *Ethanoligenens harbinense* by exogenous ethanol addition

**DOI:** 10.1186/s13068-019-1511-y

**Published:** 2019-06-28

**Authors:** Huahua Li, Xiaoxue Mei, Bingfeng Liu, Guojun Xie, Nanqi Ren, Defeng Xing

**Affiliations:** State Key Laboratory of Urban Water Resources and Environment, School of Environment, Harbin Institute of Technology, P.O. Box 2614, No. 73 Huanghe Road, Nangang District, Harbin, 150090 Heilongjiang China

**Keywords:** Hydrogen-producing bacteria, Ethanologenesis, *Ethanoligenens harbinense*, Ethanol stress, Quantitative proteomics

## Abstract

**Background:**

H_2_–ethanol-coproducing bacteria, as primary fermenters, play important roles in the microbiome of bioreactors for bioenergy production from organic wastewater or solid wastes. *Ethanoligenens harbinense* YUAN-3 is an anaerobic ethanol–H_2_-fermenting bacterium. Ethanol is one of the main end-products of strain YUAN-3 that influence its fermentative process. Until recently, the molecular mechanism of metabolic regulation in strain YUAN-3 during ethanol accumulation has still been unclear. This study aims to elucidate the metabolic regulation mechanisms in strain YUAN-3, which contributes to effectively shape the microbiome for biofuel and bioenergy production from waste stream.

**Results:**

This study reports that ethanol stress altered the distribution of end-product yields in the H_2_–ethanol-coproducing *Ethanoligenens harbinense* strain YUAN-3. Decreasing trends of hydrogen yield from 1888.6 ± 45.8 to 837 ± 64.7 mL L^−1^ and acetic acid yield from 1767.7 ± 45 to 160.6 ± 44.7 mg L^−1^ were observed in strain YUAN-3 with increasing exogenous ethanol (0 mM–200 mM). However, the ethanol yield of strain YUAN-3 increased by 15.1%, 30.1%, and 27.4% in 50 mM, 100 mM, and 200 mM ethanol stress, respectively. The endogenous ethanol accounted for 96.1% (w/w) in liquid end-products when exogenous ethanol of 200 mM was added. The molar ratio of ethanol to acetic acid increased 14 times (exogenous ethanol of 200 mM) compared to the control. iTRAQ-based quantitative proteomic analysis indicated that 263 proteins of strain YUAN-3 were differentially expressed in 50 mM, 100 mM, and 200 mM of exogenous ethanol. These proteins are mainly involved in amino acid transport and metabolism, central carbon metabolism, and oxidative stress response.

**Conclusion:**

These differentially expressed proteins play important roles in metabolic changes necessary for growth and survival of strain YUAN-3 during ethanol stress. The up-regulation of bifunctional acetaldehyde-CoA/alcohol dehydrogenase (ADHE) was the main reason why ethanol production was enhanced, while hydrogen gas and acetic acid yields declined in strain YUAN-3 during ethanol stress. This study also provides a new approach for the enhancement of ethanologenesis by H_2_–ethanol-coproducing bacteria through exogenous ethanol addition.
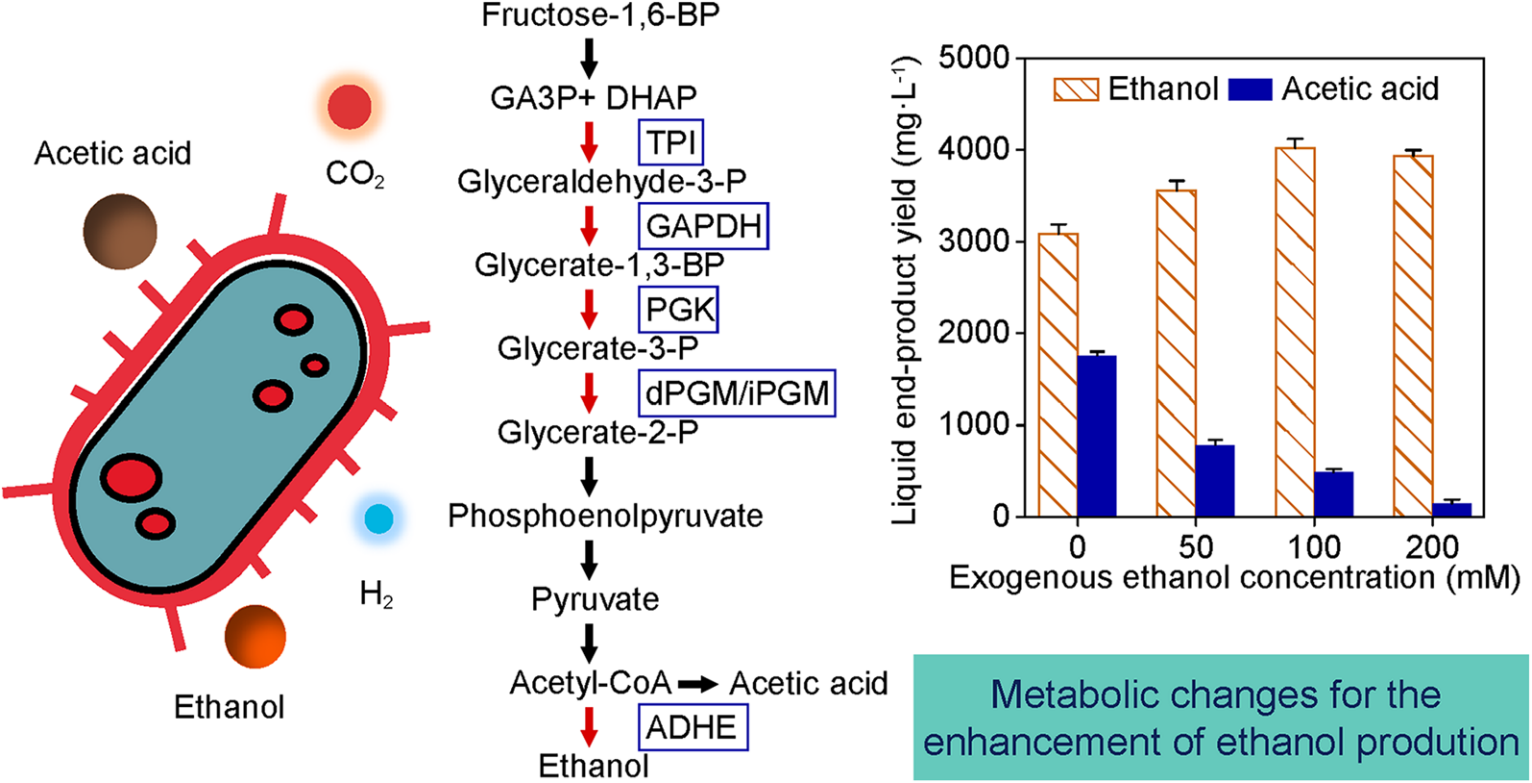

**Electronic supplementary material:**

The online version of this article (10.1186/s13068-019-1511-y) contains supplementary material, which is available to authorized users.

## Background

Microorganisms play crucial roles in biological wastewater treatment process which can effectively remove organic matters and simultaneously achieve energy recovery [1–3]. Hydrogen-producing species in activated sludge and biofilms are responsible for providing electron to methanogenesis, homoacetogenesis, and other terminal electron-accepting processes such as sulfate reduction and denitrification [4]. Gaseous hydrogen is an efficient electron carrier; recent studies revealed that hydrogen is widely used as an energy source for microbial growth and survival in biological systems. Microbial hydrogen metabolism was more widely spread than previously reported [5–7]. *Ethanoligenens harbinense* is an anaerobic ethanol–H_2_-fermenting bacterium, and the type strain YUAN-3 forms autoaggregating granules which is benefit to the formation of anaerobic granular sludge and maintenance of high cell density in continuous-flow bioreactors [8–10]. The end-products are composed of ethanol, acetic acid, H_2_, and CO_2_ [11], which can also be subsequently used by other microbial species through cross-feeding interactions in anaerobic digestion and bioelectrochemical systems (BES) to achieve higher energy recovery from organic wastes [12–14]. H_2_ and ethanol can be used directly as bioenergy and biofuel. Moreover, ethanol and acetic acid can also be converted into medium-chain fatty acids (MCFAs) by naturally present bacteria through reverse β-oxidation reaction. Additionally, MCFAs are easier to extract from water and are more versatile than ethanol and acetic acid [15, 16].

Metabolic flexibility contributes to the survival and fitness of microbial species in a changing environment [17, 18]. Decreasing pH value enhanced ethanol productivity and reduced acetic acid accumulation in *Clostridium autoethanogenum* [19]. Eight percent oxygen exposure resulted in higher ethanol yield and lower acetate yield in *Clostridium ljungdahlii* [20]. A decrease in pH is also the main factor that induces metabolic change from organic acids production to solvents production in acetone–butanol–ethanol (ABE)-fermenting bacteria [21]. Increased 3-methylbutanoic and 2-methylbutanoic acid production and reduced primary carbohydrate metabolite production were observed in *Lactobacillus sanfranciscensis* LSCE1, which is considered to be induced by acid stress [22]. Ethanol is one of the liquid end-products of strain YUAN-3 that influence its fermentative process [8]. However, the molecular mechanism of metabolic regulation in *Ethanoligenens harbinense* strain YUAN-3 during ethanol accumulation is still unclear.

End-product accumulation frequently causes inhibitory effects on cell physiology [23]. Microbial ethanol stress response has generally been described to be a complex biological process [24–26]. Ethanol stress increased membrane fluidity and denatured proteins within the cytosol and membrane, which adversely affected metabolism thus decreasing the cell growth of bacteria [27]. A previous study has proposed that translation and transcription are important processes negatively affected by ethanol. Ethanol caused the inhibition and uncoupling of mRNA and protein synthesis by directly influencing ribosome and RNA polymerase conformations in *E. coli* [28]. More recently, the effect of ethanol on the global metabolic response in *Oenococcus oeni* was carried out using an extended genome-scale metabolic model. The results indicated that the requirements of NAD(P)^+^ and ATP increased in ethanol stress, and the strain required 10 and 17 times more ATP for non-growth associated maintenance during growth in medium containing 9% and 12% ethanol, respectively [29]. Therefore, these studies demonstrated that it is difficult to determine the microbial ethanol stress response mechanisms through limited proteins or pathways.

Mass spectrometry-based proteomics has allowed an in-depth view of the proteome and extensively contributed biological insights of underlying molecular mechanisms on a global level [30, 31]. iTRAQ-based quantitative proteomics has been applied to identify and quantify proteins from a variety of prokaryotic samples simultaneously [32, 33]. Therefore, iTRAQ-based quantitative proteomic approach was conducted to examine the molecular response of strain YUAN-3 to ethanol stress. This study contributes to the elucidation of metabolic regulation mechanisms in fermentative anaerobes and improves our understanding of the cross-feeding interactions among H_2_-utilizing, acetate- and ethanol-consuming species. These information are important to effectively shape the microbiome for biofuel and bioenergy production from waste stream.

## Results and discussion

### Ethanol stress facilitates ethanologenesis and growth of strain YUAN-3

**Fig. 1:**
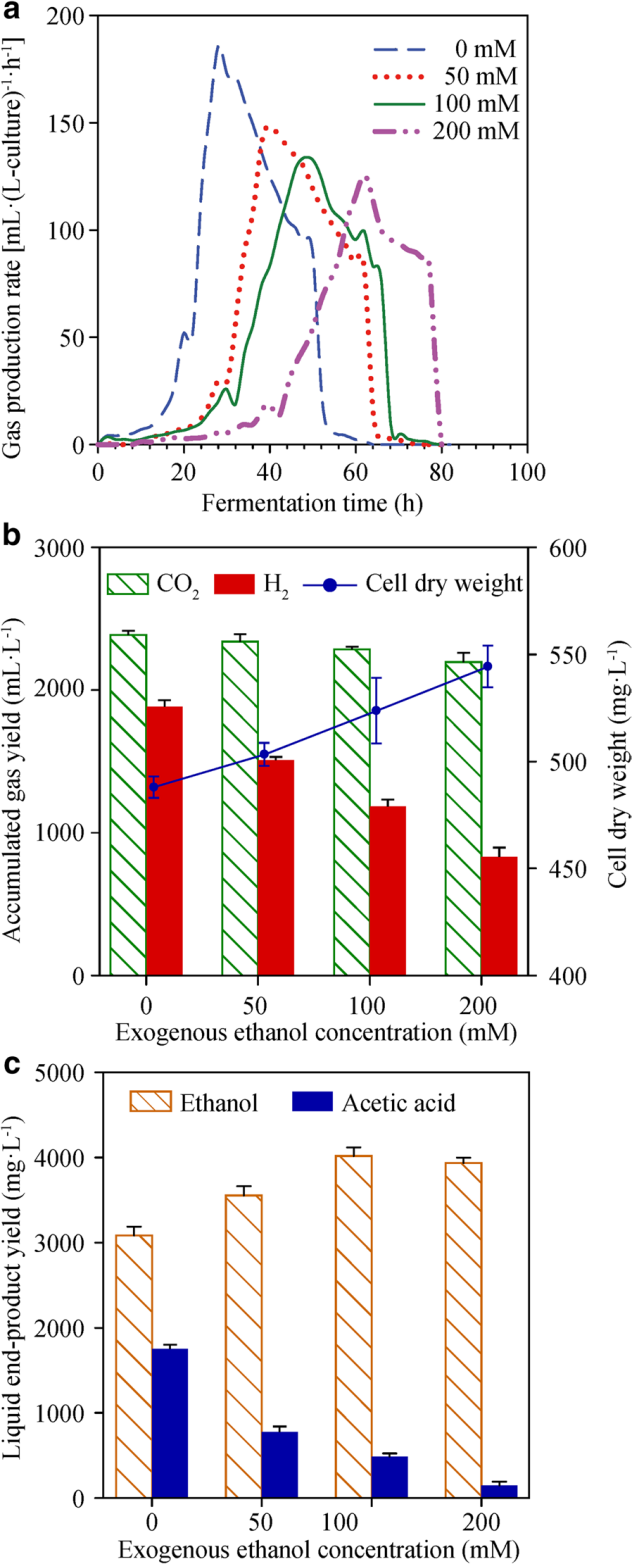
Effect of ethanol stress on end-products and cell dry weight of strain YUAN-3. **a** Gas production rate and fermentation time, **b** H_2_, CO_2_ yield, and cell dry weight, **c** ethanol and acetic acid yields

There was a significant decrease in gas production rate. The maximum gas production rate decreased from 186.05 to 125.7 mL (L-culture)^−1^ h^−1^ with increasing exogenous ethanol from 0 to 200 mM (Fig. 1a). Moreover, the fermentation time was also prolonged from 62 to 80 h with increasing exogenous ethanol (Fig. 1a). There was a clear trend of decreasing hydrogen yield from 1888.6 ± 45.8 to 837 ± 64.7 mL L^−1^ with increasing exogenous ethanol from 0 to 200 mM; however, the final cell dry weight of strain YUAN-3 increased in the presence of exogenous ethanol and the maximum cell dry weight reached 544 ± 9.7 mg L^−1^ (Fig. 1b). The acetic acid yield of strain YUAN-3 significantly decreased from 1767.7 ± 45 to 160.6 ± 44.7 mg L^−1^ in the presence of exogenous ethanol (Fig. 1c); this trend positively correlates to the hydrogen yield and negatively correlates with the final cell dry weight of strain YUAN-3 as shown in Fig. 1b. Furthermore, the endogenous ethanol yield of strain YUAN-3 increased by 15.1%, 30.1%, and 27.4% compared with control (0 mM), when the culture was supplemented with exogenous ethanol of 50 mM, 100 mM, and 200 mM, respectively. The maximum yield of endogenous ethanol reached 4030.4 ± 102.8 mg L^−1^ in the presence of 100 mM exogenous ethanol (Fig. 1c). The molar ratios of endogenous ethanol to acetic acid were 2.29, 5.88, 10.52, and 32.05 with increasing exogenous ethanol from 0 mM to 200 mM, while the weight percentage of endogenous ethanol in liquid end-products (endogenous ethanol and acetic acid) increased from 63.7 to 96.1% (w/w).

Exogenous ethanol reduced acetic acid and hydrogen production in strain YUAN-3. It could prevent a sharp decrease in pH of the medium due to acetic acid accumulation. Longer fermentation time will boost ethanol production in a continuous-flow reactor due to the mitigation of acidogenesis and substrate oxidation. However, the fermentation time of strain YUAN-3 was also prolonged by exogenous ethanol addition. Further work needs to be done, such as selecting better ethanol-tolerated strains of *Ethanoligenens harbinense*, improving the culture conditions to enhance the ethanol tolerance of strain YUAN-3.

### Identification of the differentially expressed proteins

**Fig. 2:**
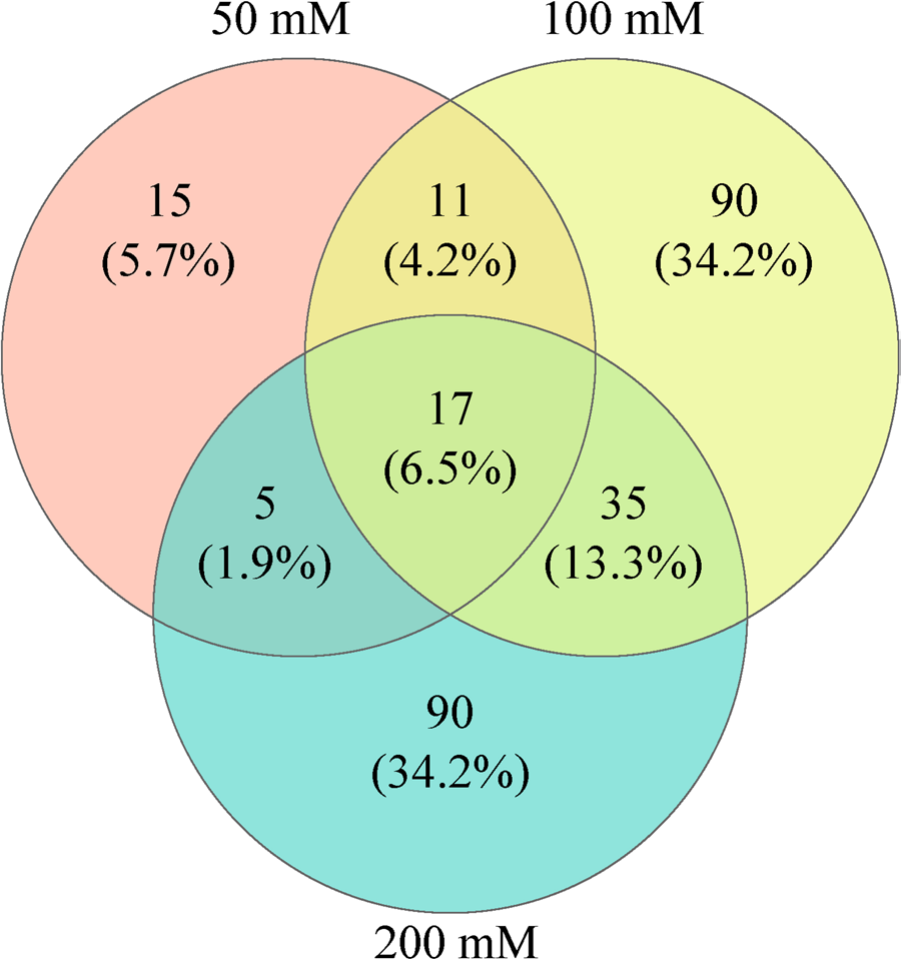
Overlap of the differentially expressed proteins of strain YUAN-3 in 50 mM, 100 mM, and 200 mM ethanol stress, respectively

**Fig. 3:**
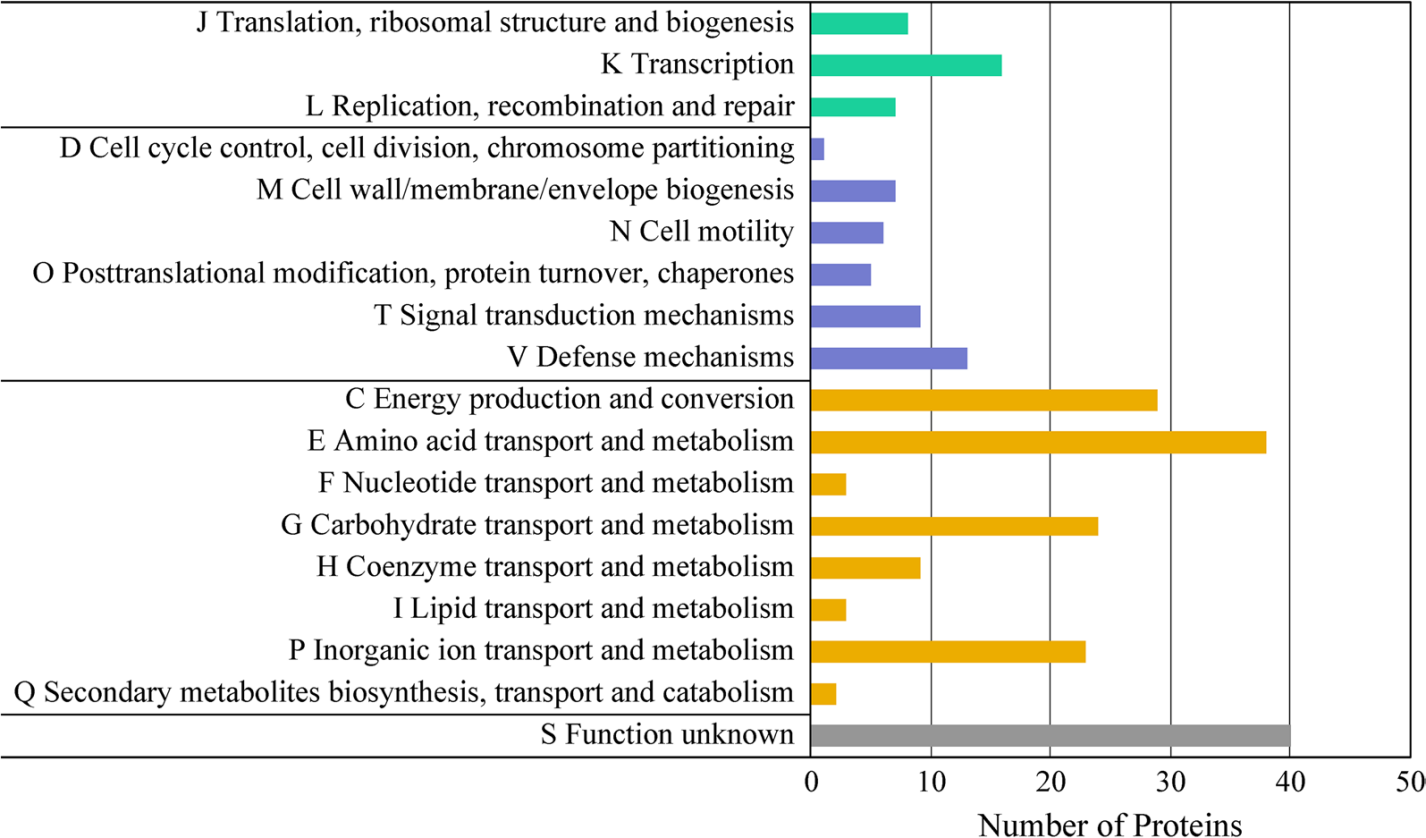
Clusters of orthologous groups (COG) classification of the differentially expressed proteins in strain YUAN-3

A total of 1680 proteins in strain YUAN-3 were identified in this study (62% of the predicted proteins in YUAN-3 proteome). A total of 1527 proteins were accurately quantified and 263 differentially expressed proteins were identified. Proteins (48, 153, and 147) were differentially expressed in the culture supplemented with exogenous ethanol of 50 mM, 100 mM, and 200 mM, respectively (Additional file 1: Tables S1, S3). A Venn diagram result showed that 90 differentially expressed proteins were exclusively grouped in 100 mM samples, which was also observed in the 200 mM samples (Fig. 2). The 263 differentially expressed proteins were further categorized into 18 groups according to clusters of orthologous groups (COG) categorization (Fig. 3). These proteins were mainly grouped into several categories, namely, amino acid transport and metabolism (38 proteins), energy production and conversion (29 proteins), carbohydrate transport and metabolism (24 proteins), inorganic ion transport and metabolism (23 proteins), and transcription (16 proteins). Moreover, 40 proteins belong to the unknown function group, while 20 proteins share no significant similarity with any group.

### Functions of the differentially expressed proteins in ethanol stress

**Fig. 4:**
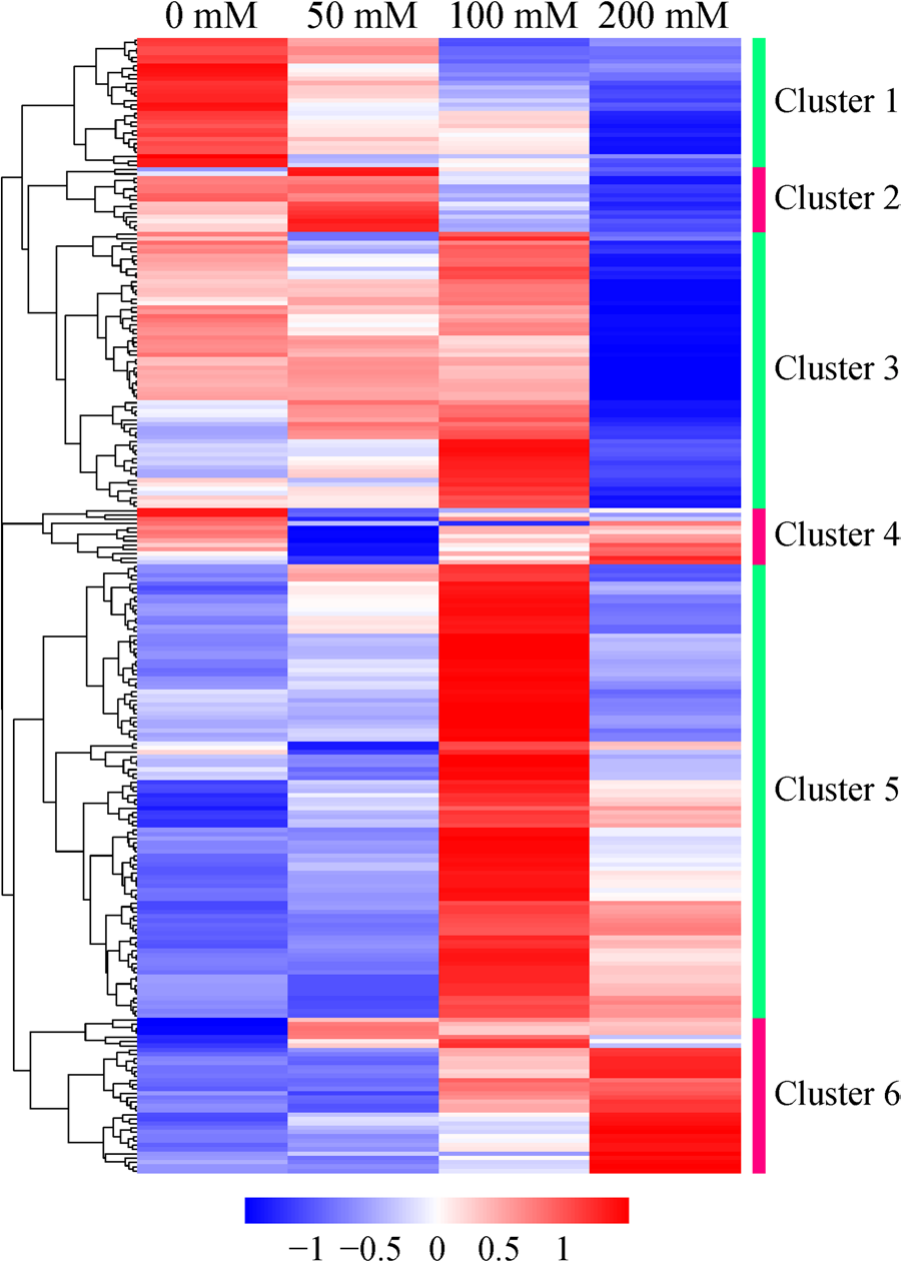
Heatmap of the differentially expressed proteins in strain YUAN-3. The blue color represents lower z-scores indicating lower relative abundances for each protein. A z-score of 0 represents a protein abundance value that is equal to the average abundance value of all abundances for that given protein

The clustering analysis was performed based on similar expression profiles of proteins. The 263 differentially expressed proteins were classified into 6 major clusters and the result was displayed in a heatmap (Fig. 4). To understand the functions of these proteins better, all the proteins in each cluster were further analyzed according to the KEGG pathway enrichment. The results revealed that ethanol caused dramatic changes in the protein expression profiles of strain YUAN-3 (Additional file 1: Table S4). Thirty proteins were classified into cluster 1; these proteins were mainly involved in ABC transporters, phosphotransferase system (PTS), fructose and mannose metabolism. The expression levels of proteins in cluster 1 were down-regulated in the presence of ethanol, thus indicating that the expression levels of these proteins were negatively related to the ethanol concentrations. Fifteen proteins were grouped in cluster 2 and the expression levels of most proteins in this cluster were down-regulated at 100 mM exogenous ethanol concentration. The expression levels of proteins which participate in riboflavin metabolism (vitamin B2) in this cluster were up-regulated at 50 mM exogenous ethanol concentration. Sixty-four proteins were classified into cluster 3; the expression levels of these proteins were down-regulated when the exogenous ethanol concentration reached 200 mM. Proteins in this cluster are mainly associated with oxidative phosphorylation, two-component system, and flagellar assembly. Thirteen proteins were grouped in cluster 4. The expression levels of these proteins were down-regulated at 50 mM exogenous ethanol concentration. The proteins in this cluster mainly participate in the phosphotransferase system (PTS). One hundred and five proteins were classified into cluster 5 and the expression levels of these proteins were elevated at 100 mM exogenous ethanol concentration. Proteins in this cluster are mainly involved in histidine metabolism, biosynthesis of amino acids, biosynthesis of secondary metabolites, and glycolysis. Thirty-six proteins were grouped in cluster 6. The expression levels of most proteins in this cluster were up-regulated in the presence of ethanol. The expression levels of these proteins were positively related to the ethanol concentrations. Proteins in cluster 6 are mainly associated with microbial metabolism in diverse environments and nitrogen metabolism.

### Exogenous ethanol up-regulated ethanologenesis-related proteins

**Fig. 5:**
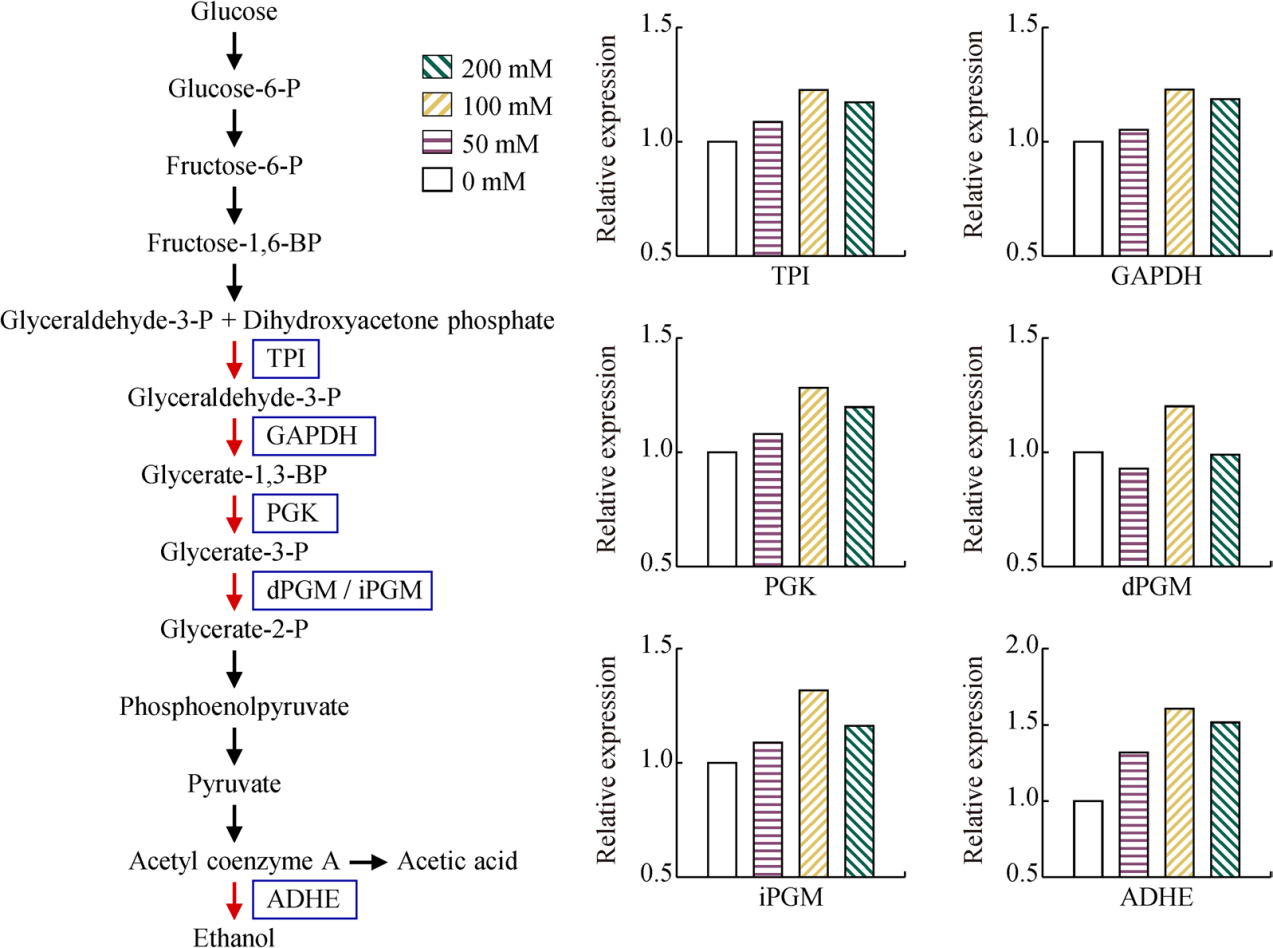
The differentially expressed proteins involved in glycolysis and ethanol production of strain YUAN-3. TPI, triosephosphate isomerase; GAPDH, glyceraldehyde-3-phosphate dehydrogenase; PGK, phosphoglycerate kinase; dPGM, 2, 3-diphosphoglycerate-dependent phosphoglycerate mutase; iPGM, 2, 3-bisphosphoglycerate-independent phosphoglycerate mutase; ADHE, bifunctional acetaldehyde-CoA/alcohol dehydrogenase

Bifunctional acetaldehyde-CoA/alcohol dehydrogenase (ADHE) has been demonstrated to be a key enzyme in ethanol production. This enzyme contains two catalytic reaction domains which are responsible for the conversion of acetyl-coenzyme A to ethanol. The N-terminal acetaldehyde dehydrogenase (ALDH) domain of ADHE is responsible for the conversion of acetyl-CoA into acetaldehyde; while the C-terminal alcohol dehydrogenase (ADH) domain of ADHE is responsible for the conversion of acetaldehyde to ethanol [34]. ADHE (ADU26923) of strain YUAN-3 was identified with 10,944 peptide–spectrum matches which is the second most numerous peptide–spectrum matches of all the identified proteins in strain YUAN-3. The data suggested that ADHE was one of the most abundant proteins in strain YUAN-3 (Additional file 1: Table S5). Surprisingly, we also found that ADHE exhibited 1.32-, 1.61-, and 1.52-fold change in the presence of 50 mM, 100 mM, and 200 mM exogenous ethanol, respectively. Thus, the expression level of ADHE was observed to be closely related to the endogenous ethanol yields that exhibited 1.15-, 1.30-, and 1.27-fold change, respectively (Figs. 1c and 5). This result clearly demonstrates that the endogenous ethanol yield of strain YUAN-3 increases when exogenous ethanol is added to the medium (Fig. 1c). Although ethanol yield of *Ethanoligenens* is lower than yeast, this genus can simultaneously produce ethanol of high purity (96.1%, w/w) and hydrogen gas. In contrast, yeast cannot produce hydrogen gas that has much higher combustion efficiency than ethanol. H_2_ and ethanol co-production makes this *Ethanoligenens* become a promising candidate for production of biofuel and bioenergy from wastewater.

In addition, the syntheses of ethanol and acetic acid in *Ethanoligenens* are acetyl-CoA-consuming reactions that directly compete for acetyl-CoA. On the other hand, the productions of ethanol and hydrogen gas are NADH-consuming reactions that compete for NADH. Therefore, increased yield of ethanol resulted in lower conversion of acetyl-CoA into acetic acid and also lower NADH for hydrogen production (Fig. 1b, c). This could explain why the yields of hydrogen gas and acetic acid declined even though expression levels of [FeFe]-hydrogenase (H_2_ase) and acetate kinase did not show significant change correspondingly.

We found five enzymes related to the glycolysis pathway that were up-regulated at 100 mM ethanol stress and grouped in cluster 5 (Figs. 4 and 5). These glycolytic enzymes include phosphoglycerate kinase (PGK, ADU27083), triosephosphate isomerase (TPI, ADU27084), glyceraldehyde-3-phosphate dehydrogenase (GAPDH, ADU28097), 2, 3-diphosphoglycerate-dependent phosphoglycerate mutase (dPGM, ADU26920), and 2, 3-bisphosphoglycerate-independent phosphoglycerate mutase (iPGM, ADU27085). Certain eubacteria possess both dPGM and iPGM, but dPGM has a tenfold higher specific activity than iPGM to catalyze the interconversion of 2-phosphoglycerate and 3-phosphoglycerate in glycolysis [35]. The expression levels of these glycolytic enzymes and the endogenous ethanol yield of strain YUAN-3 are tightly related (Fig. 1c). Accumulated evidence showed that cells must expend considerably high levels of energy to mitigate ethanol stress. Cells repair cellular macromolecules that were damaged in ethanol stress and employ efflux pumps for dealing with ethanol toxicity [36]. Therefore, the up-regulated expression levels of glycolytic enzymes in strain YUAN-3 suggest that ethanol stress increases the demand for energy to increase tolerance. In addition, increased expression levels of glycolytic enzymes in the presence of 100 mM exogenous ethanol would contribute to the production of NADH and pyruvate which increases the production of ethanol in strain YUAN-3. This could explain why strain YUAN-3 achieved the highest endogenous ethanol yield in the presence of 100 mM exogenous ethanol compared to other samples in this study (Fig. 1c).

Both ethanol synthesis and hydrogen production are NADH-consuming reactions which further influence the cellular NADH/NAD^+^ levels. The redox-sensing transcriptional repressor Rex plays a key role in sensing cellular NADH/NAD^+^ levels and it negatively controls the transcription of a large variety of NADH/NAD^+^-utilizing redox enzymes which influence the cellular NADH/NAD^+^ balance. Under the condition of low cellular NADH/NAD^+^ ratio, Rex binds to the target DNA sites and represses transcription of target genes [37]. Through comparative genomics approach analysis, results indicate that Rex target genes are mainly involved in energy metabolism, central carbohydrate metabolism, fermentation pathways, nitrate/nitrite and sulfate/sulfite reduction pathways, and NAD(P)H biogenesis pathways [38]. In addition, the novel function of Rex in the control of hydrogen production genes was validated in hydrogen-producing bacterium *Thermotoga maritima* [38]. In this study, we found the endogenous ethanol production yield of strain YUAN-3 increased; while the hydrogen product yield decreased in the presence of exogenous ethanol (Fig. 1b, c). According to iTRAQ analysis, the expression level of redox-sensing transcriptional repressor Rex (ADU26924) was up-regulated in the presence of 100 mM exogenous ethanol (Additional file 1: Table S2). Thus, we speculate that the Rex-dependent regulation system regulates ethanologenesis and hydrogen production of strain YUAN-3, thus subsequently altering the yield of each end-product.

The cell dry weight of strain YUAN-3 increased in the presence of exogenous ethanol (Fig. 1b). An efficient biomass formation resulted into less amount of substrate transformed into end-products; thus, this result could partially explain why the yields of hydrogen gas and acetic acid declined in this study. More recently, the effect of ethanol on the global metabolic response in *Oenococcus oeni* was investigated using an extended genome-scale metabolic model. The results indicated that the requirements of NAD(P)^+^ and ATP increased during ethanol stress. The strain required 10 and 17 times more non-growth associated maintenance ATP during growth in medium containing 9% and 12% ethanol, respectively [29]. Therefore, the altered distribution of end-product yields in strain YUAN-3 indicates the need to redirect energy flow to maintain an efficient balance between ethanol stress response and growth.

### Proteins involved in carbon and nitrogen metabolism

According to COG categorization, we observed that 38 proteins were involved in the group of amino acid transport and metabolism (Fig. 3), which has the highest number of differentially expressed proteins in strain YUAN-3 compared to other COG groups. This result is consistent with the response mechanism of *Clostridium thermocellum* ATCC27405 to ethanol stress, in which proteins related to nitrogen uptake and metabolism are most affected [39]. In addition, 24 proteins were found in the group of carbohydrate transport and metabolism (Fig. 3). These results indicated that carbon and nitrogen metabolism is a crucial process for dealing with ethanol stress in strain YUAN-3.

Proteins of strain YUAN-3 involved in nitrogen metabolism were differentially expressed in ethanol stress. Four proteins involved in urea cycle and metabolism were significantly up-regulated in 100 mM ethanol stress. Two of these proteins were up-regulated in 50 mM ethanol stress. These proteins were grouped into cluster 5 (Fig. 4 and Additional file 1: Tables S1, S2) including urea carboxylase (ADU27908), allophanate hydrolase (ADU27909), and urea carboxylase-associated protein (ADU27906 and ADU27907). A recent study has revealed that the urea carboxylase of *Oleomonas sagaranensis* can also use guanidine as substrates. Guanidine in bacteria can be degraded in the same manner as urea but the catalytic efficiency was observed to be 40 times better for guanidine than urea [40]. They also demonstrated that bacteria are capable of endogenously producing guanidine, which is the ligand of guanidine-I riboswitches and induces the expression of guanidine-I riboswitch-mediated genes [40]. The guanidine-I riboswitches were reported to control the expression of a variety of transporter and metabolic genes, including genes annotated as urea carboxylases, urea carboxylase-associated protein, allophanate hydrolase, nitrate/sulfate/bicarbonate transporters, multidrug resistance transporters, and genes involved in nitrogen metabolism [41, 42]. We also identified two proteins (ADU27903 and ADU27905) of ABC-type nitrate/sulfonate/bicarbonate transport system that were up-regulated in 50 mM and 100 mM ethanol stress (Additional file 1: Tables S1, S2). Since urea was not provided as nitrogen source in the medium and homologue enzymes that catalyze arginine to ornithine and urea have not been identified in strain YUAN-3; it is possible that ethanol stress increases the cellular concentration of guanidine in strain YUAN-3. Ethanol stress subsequently induces the expression of guanidine-I riboswitch-mediated genes.

The carbon storage regulator protein (CsrA) has been known to control a number of physiological processes such as central carbon metabolism, acetate metabolism, stress response, biofilm formation, flagellum biosynthesis, and peptide uptake [43]. It has been observed to positively regulate glycolysis, flagellum biosynthesis, and acetate metabolism [43, 44]. In this study, the expression level of CsrA (ADU28042) was down-regulated in the presence of 200 mM ethanol stress (Additional file 1: Table S3). Since CsrA positively regulates glycolysis, decreased expression of CsrA would result in the repression of glycolysis. The result could partially explain why the batch fermentation time of strain YUAN-3 was longer in 200 mM ethanol stress than other samples (Fig. 1a). Six proteins involved in flagellum biosynthesis including flagellar motor protein MotA (ADU28031), flagellar export chaperone FliS (ADU28033), flagellin (ADU28041), flagellar motor protein MotP (ADU28061), flagellar hook-basal body protein (ADU28063), and flagellar protein export ATPase FliI (ADU28068) were also down-regulated in 200 mM ethanol stress (Additional file 1: Table S3). These proteins and CsrA belong to cluster 3 (Fig. 4). These results support previous studies that CsrA positively regulates flagellum biosynthesis. Furthermore, CsrA is also associated with the stress response. The CsrA of *Clostridium beijerinckii* NCIMB 8052 was reported significantly repressed in furfural stress [45]. Thus, the results of this study also suggest that CsrA plays an important role in ethanol stress response.

### Proteins involved in the tolerance of ethanol stress

In this study, we observed that two oxidative stress response proteins were induced during ethanol stress, which includes desulfoferrodoxin (ADU28196) and glutathione peroxidase (ADU28264) (Additional file 1: Tables S2, S3). Desulfoferrodoxin has been reported to function as a superoxide reductase (SOR) and superoxide dismutase (SOD), which protects anaerobic bacteria from oxidative stress [46, 47]. Through DNA microarray analysis, the result has shown that desulfoferrodoxin gene expression of *Clostridium acetobutylicum* was up-regulated in butanol, butyrate, and acetate stress [48]. The main biological role of glutathione peroxidase is to protect the organism from oxidative stress. Previous research has reported that glutathione peroxidase of *Lactobacillus plantarum* WCFS1 was induced during growth while exposed to ethanol stress [49]. In this study, desulfoferrodoxin of strain YUAN-3 was found to be up-regulated in 100 mM and 200 mM ethanol stress; while glutathione peroxidase was up-regulated in 100 mM ethanol stress. These results indicate new potential protective roles of desulfoferrodoxin and glutathione peroxidase in ethanol stress response.

**Fig. 6:**
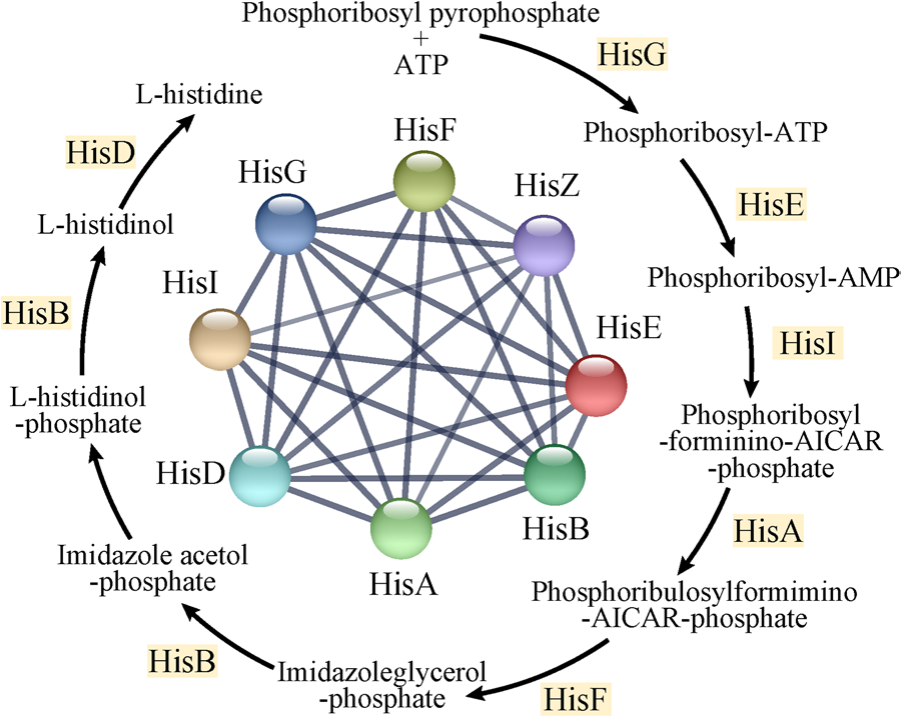
Protein–protein interaction network of the differentially expressed proteins involved in histidine biosynthesis in strain YUAN-3. The network nodes are proteins; the thickness of the network line indicate the degree of confidence prediction of the interaction. The minimum required interaction score is 0.4. AICAR, 5′-phosphoribosyl-4-carboxamide-5-aminoimidazole; HisG, ATP phosphoribosyltransferase; HisE, phosphoribosyl-ATP pyrophosphatase; HisI, phosphoribosyl-AMP cyclohydrolase; HisA, 1-(5-phosphoribosyl)-5-[(5-phosphoribosylamino) methylideneamino] imidazole-4-carboxamide isomerase; HisF, imidazole glycerol phosphate synthase subunit; HisB, imidazoleglycerol-phosphate dehydratase; HisD, histidinol dehydrogenase; HisZ, ATP phosphoribosyltransferase regulatory subunit

Eight proteins of strain YUAN-3 involved in histidine biosynthesis were also identified to be up-regulated in 100 mM ethanol stress; three of these proteins were up-regulated in 200 mM ethanol stress (Fig. 6 and Additional file 1: Tables S2, S3). These proteins belong to cluster 5 (Additional file 1: Table S4), including HisE (ADU26970), HisI1 (ADU26971), HisF (ADU26972), HisA (ADU26973), HisB (ADU26975), HisD (ADU26977), HisG (ADU26978), and HisZ (ADU26979). It has been shown that increased expression of genes for histidine biosynthesis contributes to acid tolerance in *Lactobacillus casei*. The strain displayed a 100-fold increase in survival after exogenous addition of histidine to the medium during acid stress [50]. Nine histidine biosynthesis genes of *Clostridium acetobutylicum* were up-regulated in butanol and butyrate stress [51]. Up-regulation of histidine biosynthesis genes was also observed in ethanol tolerant *Escherichia coli* strains [52]. Therefore, it is plausible that histidine biosynthesis elevates the ethanol tolerance of strain YUAN-3.

### Perspective and outlook

Proteins directly participate in the biological processes and functions; thus, the quantitative proteomic analysis can provide direct evidences for the regulation mechanisms of physiology and metabolism at global level [53]. Because the correlation between transcriptome (mRNA) and proteome is often low, quantitative proteomic analysis becomes especially important to reveal the regulation mechanism of biological processes [54]. This study gave an example to reveal the regulation of H_2_–ethanol fermentative metabolism based on quantitative proteomic analysis, and the experimental protocol can be used to investigate the metabolic regulation and function of other microbes. These results indicated that the protein regulation of anaerobes was complicated and interactive. Biological processes usually involve multiple proteins, the amount and variety of these proteins are unknown. Therefore, it is difficult to reveal the mechanism of metabolic regulation through conventional approach that focuses on single or few target proteins. The hydrogenase expression level showed no significant change even if hydrogen production changed in strain YUAN-3. The regulation of hydrogen production may be directly dependent upon the activity of hydrogenase, and the upstream proteins responsible for electron transfer and NADH generation in hydrogen production pathway. Besides, further investigations need to be done on protein post-translational modification and protein–protein interaction of the enzymes which were not significantly changed.

## Conclusions

Exogenous ethanol altered the yields of end-products in *E. harbinense* strain YUAN-3. Ethanol stress enhanced ethanol production and also inhibited the production of hydrogen gas and acetic acid. Differentially expressed proteins (48, 153, and 147) were identified in 50 mM, 100 mM, and 200 mM ethanol stress, respectively. These proteins are mainly involved in central carbon metabolism, amino acid transport and metabolism, and oxidative stress response. The distribution of end-product yields was altered during ethanol stress in strain YUAN-3 due to the up-regulation of ethanologenesis-related proteins by exogenous ethanol. The differentially expressed proteins also corresponded to metabolic changes necessary to the growth and survival of *E. harbinense* YUAN-3 during ethanol stress. This study also provided a new approach to change the pattern of end-products and enhance ethanol production by *Ethanoligenens harbinense* through exogenous ethanol addition. This approach can be easily employed via the circulation of liquid end-products in a continuous-flow anaerobic bioreactor. *Ethanoligenens* has been identified as a predominant population in ethanol-type fermentation, which becomes one of the representative hydrogen-producing genera. Therefore, the ethanol production in ethanol-type fermentation of mixed cultures could be enhanced through this approach. However, this regulatory strategy must be further verified in the continuous-flow anaerobic bioreactors.

## Methods

### Culture conditions

*Ethanoligenens harbinense* strain YUAN-3 was cultivated in anaerobic PYG medium at 35 °C with initial pH value of 7. The composition of 1 L PYG medium include glucose 10 g, peptone 4 g, yeast extract 1 g, NaCl 4 g, K_2_HPO_4_ 1.5 g, MgCl·6H_2_O 0.1 g, and L-cysteine 0.24 g. Mineral salt solution and vitamin solution were added according to the previous description [55]. In the experiment investigating effect of ethanol stress on end-products and cell dry weight of strain YUAN-3, 4 mL (2%, v/v) of logarithmic stage YUAN-3 culture was used to inoculate into 200-mL medium. Different final concentrations (50, 100, 200 mM) of exogenous ethanol were added to the medium before the experiment. The medium without ethanol served as the control.

### End-products and accumulated biomass analysis

Challenge AER-208 Aerobic/Anaerobic Respirometer System (Challenge Environmental System, Arkansas) was used to investigate the gas yield and production rate of strain YUAN-3. H_2_, CO_2_, alcohols, volatile fatty acids and biomass were analyzed after the fermentation process ended. H_2_ and CO_2_ were determined using gas chromatograph (Agilent GC7890A, USA) equipped with thermal conductivity detector, while alcohols and volatile fatty acids were analyzed via gas chromatography (Agilent GC7890A, USA) equipped with flame ionization detector [56]. All the ethanol in each YUAN-3 culture was analyzed by gas chromatography, respectively; the endogenous ethanol yields were calculated by deducting the amount of exogenous ethanol. For cell dry weight measurement, YUAN-3 cultures were centrifuged at 10,000×*g* for 10 min at 4 °C. The pellets were washed twice in PBS buffer and dried at 105 °C.

### Sample preparation and protein extraction for proteomic analysis

To guarantee that ethanol is the single factor that affects metabolism of strain YUAN-3, the medium of YUAN-3 culture in logarithmic stage (100 mL) was removed by centrifugation at 10,000×*g* for 5 min; the YUAN-3 pellets were immediately transferred to 200-mL fresh medium with exogenous ethanol at anaerobic conditions. Exogenous ethanol was supplemented to the medium at different final concentrations 0, 50, 100, and 200 mM, respectively. The YUAN-3 culture was cultivated at 35 °C for 2 h and collected by centrifugation (10,000×*g*, 5 min) at 4 °C. There were 2.92, 2.99, 3.36, 3.55 mM (134.7, 137.6, 154.9, 163.3 mg L^−1^) ethanol endogenously generated by strain YUAN-3 at 2 h with increasing exogenous ethanol from 0 mM to 200 mM, respectively, which were trace amount compared with the exogenous ethanol, and can be neglected in this study. The pellets were washed twice with chilled anaerobic PBS buffer (pH 7.4, NaCl 137 mM, KCl 2.7 mM, Na_2_HPO_4_ 10 mM, KH_2_PO_4_ 2 mM). FastPrep-24 instrument (MP Biomedical) was used for homogenization; 30 mg sample was transferred to lysing matrix B tubes with 500 µL chilled Tris pH 8.8 buffered phenol (Sigma) and 500 µL chilled extraction buffer. The samples were homogenized twice for 40 s and chilled on ice for 3 min between each cycle. The extraction buffer contained 0.1 M Tris–HCl (pH 8.8), 10 mM EDTA, 0.9 M sucrose, 0.4% (v/v) 2-mercaptoethanol, and 1% (v/v) protease inhibitor cocktail (set II, Calbiochem). The following steps were performed according to phenol extraction method as previously described [57].

### Protein digestion and iTRAQ labeling

The biological duplicate protein samples (0.75 mg) were reconstituted in 150 µL 1 × LDS (Invitrogen) with 50 mM DTT (dithiothreitol), followed by incubation at 90 °C for 10 min. Ten percent of each sample were loaded and run into SDS-PAGE gel. The in-gel protein samples were reduced with 10 mM DTT at 60 °C for 30 min and alkylated with 20 mM iodoacetamide at room temperature and dark conditions for 1 h. The samples were digested overnight with trypsin (1/50, w/w, trypsin/sample) at 37 °C. The digested peptides were subsequently extracted from the SDS-PAGE gel with buffer (60% acetonitrile, 5% formic acid) and vacuum-dried to remove residual amine from the reagents. Peptides were labeled with iTRAQ 4-plex Kit (ABSciex, USA) according to the manufacturer’s protocol and then combined after labeling.

### Nano-LC–MS/MS and bioinformatics analysis

The Nano-LC–MS/MS analysis was carried out using a Dionex RSLC system coupled to Q-Exactive HF mass spectrometer (ThermoFisher, San Jose, CA). The prepared samples were dissolved in 0.1% trifluoroacetic acid and loaded into a home-made trap (100 µm × 2 cm, packed with Magic C18AQ, 5 µm, 200 Å pore size: Michrom Bioresources, Inc., Auburn, CA). Solvent A (0.2% formic acid) was used to wash the samples at a flow rate of 10 µL min^−1^ for 5 min. The trap column was then connected to a homemade analytical column (Magic C18AQ, 3 µm, 200 Å pore, 75 µm × 50 cm). Peptide fractions were separated at a flow rate of 300 nL min^−1^ using a multi-stepped gradient of solvent B (0.16% formic acid and 80% acetonitrile): 4–15% solvent B for 25 min, 15–25% solvent B for 65 min, 25–50% solvent B for 55 min. The data-dependent acquisition procedure was performed to acquire mass spectrometry data. A full MS survey scan was recorded in the Orbitrap MS at a resolution of 120,000. The twenty most intense ions were isolated and analyzed subsequently in the Orbitrap at a resolution of 30,000. The relative collision energy was set to 30% in the HCD and dynamic exclusion duration was employed for 30 s during the subsequent MS/MS scans.

The Nano-LC–MS/MS data were searched in MUDPIT style against the protein database of *Ethanoligenens harbinense* YUAN-3 (http://bacteria.ensembl.org/Ethanoligenens_harbinense_yuan_3/Info/Index/; genome assembly: ASM17811v2) using an inhouse version of X!Tandem Sledgehammer (http://www.thegpm.org/tandem). The search parameters include the following: carbamidomethylation on cysteine and iTRAQ 4-plex label on lysine as fixed modifications, while iTRAQ 4-plex label on N-terminus of peptides and oxidation of methionine served as variable modification. Tolerance for precursor was ± 7 ppm and product ions was 20 ppm and false discovery rate (FDR) ≤ 1%. Intensity of iTRAQ 4-plex reporter ions of each spectrum was extracted by inhouse Perl script and corrected for isotope cross-over according to the values supplied by manufacturer. The ratio between samples was calculated based on reporter ion intensity and normalized to ratio of summed ion intensity of all identified spectra that fit certain criteria: peptides has iTRAQ 4-plex label, peptide belongs to *Ethanoligenens harbinense* YUAN-3 protein database, peptide log(*e*) ≤ − 2.0. Spectra that have ratio as “Divide 0” were replaced with an arbitrary number “10”. Pairwise median ratios of individual protein were calculated by “Doby” package under R environment using all spectra belonging to a protein that fit the criteria described above plus the requirement that the sum of reporter ion intensity of both channels > 20,000. Peptide–spectrum matches ≥ 3 and *P* ≤ 0.01 were required for protein quantitation.

The differentially expressed proteins were defined according to previous studies (fold change of ≤ 0.83 for down-regulated proteins, ≥ 1.2 for up-regulated proteins) and manually annotated through NCBI non-redundant protein sequences database (https://blast.ncbi.nlm.nih.gov/Blast.cgi?PROGRAM=blastp&PAGE_TYPE=BlastSearch&LINK_LOC=blasthome) and UniProt protein database (https://www.uniprot.org/blast/) with local BLAST programs (*E*-value ≤ 1.0E−5) [53, 58]. The heatmap was generated using the pheatmap package of version 3.2.3 on the R statistical platform (https://CRAN.R-project.org/package=pheatmap) KEGG database and KOBAS 3.0 (http://kobas.cbi.pku.edu.cn/) were used to carried out the pathway enrichment of the differentially expressed proteins; pathways with *P* value of ≤ 0.05 were considered significantly enriched [59]. The protein–protein interaction analysis was conducted with STRING database of version 10.5 (http://string-db.org).

## Additional file

**Additional file 1: Table S1.** The differentially expressed proteins of strain YUAN-3 in 50 mM ethanol stress. **Table S2.** The differentially expressed proteins of strain YUAN-3 in 100 mM ethanol stress. **Table S3.** The differentially expressed proteins of strain YUAN-3 in 200 mM ethanol stress. **Table S4.** KEGG pathway enrichment of the differentially expressed proteins in strain YUAN-3. **Table S5.** The ten most numerous peptide–spectrum matches of all the identified proteins in strain YUAN-3.

## Data Availability

The datasets used and/or analyzed during the current study are available from the corresponding author on reasonable request.

## Abbreviations

iTRAQ: isobaric tags for relative and absolute quantification
BES: bioelectrochemical systems
MCFAs: medium-chain fatty acids
ABE-fermenting bacteria: acetone–butanol–ethanol-fermenting bacteria
COG: clusters of orthologous groups
PTS: phosphotransferase system
ADHE: bifunctional acetaldehyde-CoA/alcohol dehydrogenase
ALDH: acetaldehyde dehydrogenase
ADH: alcohol dehydrogenase
PGK: phosphoglycerate kinase
TPI: triosephosphate isomerase
GAPDH: glyceraldehyde-3-phosphate dehydrogenase
dPGM: 2, 3-diphosphoglycerate-dependent phosphoglycerate mutase
iPGM: 2, 3-bisphosphoglycerate-independent phosphoglycerate mutase
CsrA: carbon storage regulator protein
AICAR: 5′-phosphoribosyl-4-carboxamide-5-aminoimidazole
HisG: ATP phosphoribosyltransferase
HisE: phosphoribosyl-ATP pyrophosphatase
HisI: phosphoribosyl-AMP cyclohydrolase
HisA: 1-(5-phosphoribosyl)-5-[(5-phosphoribosylamino) methylideneamino] imidazole-4-carboxamide isomerase
HisF: imidazole glycerol phosphate synthase subunit
HisB: imidazoleglycerol-phosphate dehydratase
HisD: histidinol dehydrogenase
HisZ: ATP phosphoribosyltransferase regulatory subunit
EDTA: ethylenediaminetetraacetic acid
DTT: dithiothreitol
SDS-PAGE: sodium dodecyl sulfate-polyacrylamide gel electrophoresis
FDR: false discovery rate
