# Supplementary material for: Quantitative proteomic analysis reveals the ethanologenic metabolism regulation of Ethanoligenens harbinense by exogenous ethanol addition
Source: Biotechnol Biofuels. 2019 Jun 28;12:166. doi: 10.1186/s13068-019-1511-y (PMC6598285; doi:10.1186/s13068-019-1511-y)
Supplement: Supplementary file 1 — Additional file 1: Table S1. The differentially expressed proteins of strain YUAN-3 in 50 mM ethanol stress. Table S2. The differentially expressed proteins of strain YUAN-3 in 100 mM ethanol stress. Table S3. The differentially expressed proteins of strain YUAN-3 in 200 mM ethanol stress. Table S4. KEGG pathway enrichment of the differentially expressed proteins in strain YUAN-3. Table S5. The ten most numerous peptide–spectrum matches of all the identified proteins in strain YUAN-3. [file 13068_2019_1511_MOESM1_ESM.docx]

**Additional Material**

**Quantitative proteomic analysis reveals the ethanologenic metabolism regulation of *Ethanoligenens harbinense* by exogenous ethanol addition**

**Huahua Li, Xiaoxue Mei, Bingfeng Liu, Guojun Xie, Nanqi Ren, Defeng Xing**^*^

State Key Laboratory of Urban Water Resource and Environment, School of Environment, Harbin Institute of Technology, Harbin 150090, China

^*^ **Corresponding author**. School of Environment, Harbin Institute of Technology, P.O. Box 2614, No. 73 Huanghe Road, Nangang District, Harbin, Heilongjiang Province 150090, China

E-mail address: [dxing@hit.edu.cn](mailto:dxing@hit.edu.cn)

Tel& Fax: (+86) 451 8628123

**Table S1.** The differentially expressed proteins of strain YUAN-3 in 50 mM ethanol stress

| Protein name | Fold  change | Acession  number | Functional description | Coverage | Peptides | Peptide-  spectrum  matches |
| --- | --- | --- | --- | --- | --- | --- |
|  |  |  | **No COG ID available** |  |  |  |
| hypothetical protein | 0.83 | ADU25801 |  | 10.5% | 1 | 5 |
| hypothetical protein | 1.41 | ADU27017 |  | 27.8% | 1 | 3 |
| hypothetical protein | 0.83 | ADU27745 |  | 14.3% | 1 | 4 |
| hypothetical protein | 0.81 | ADU27816 |  | 27.8% | 3 | 3 |
|  |  |  | **Information storage and processing** |  |  |  |
| ribosomal protein S6 | 0.82 | ADU28007 | J Translation, ribosomal structure and biogenesis | 80.0% | 8 | 225 |
| transcriptional regulator, MarR family | 1.29 | ADU26147 | K Transcription | 1.6% | 1 | 6 |
| transcriptional regulator, GntR family | 1.23 | ADU27755 | K Transcription | 67.6% | 18 | 178 |
|  |  |  | **Cellular processes and signaling** |  |  |  |
| ABC transporter permease | 0.75 | ADU25916 | V Defense mechanisms | 47.1% | 26 | 145 |
| ABC transporter ATP-binding protein | 0.69 | ADU25917 | V Defense mechanisms | 64.3% | 14 | 168 |
| ABC transporter permease | 1.34 | ADU26693 | V Defense mechanisms | 12.2% | 3 | 11 |
| protein of unknown function DUF214 | 0.83 | ADU26943 | V Defense mechanisms | 4.3% | 2 | 3 |
| ABC-type multidrug transport system, ATPase and permease component | 1.36 | ADU27717 | V Defense mechanisms | 1.4% | 1 | 3 |
| MATE family efflux transporter | 0.83 | ADU28252 | V Defense mechanisms | 1.7% | 1 | 3 |
|  |  |  | **Metabolism** |  |  |  |
| Aldehyde Dehydrogenase | 1.22 | ADU26155 | C Energy production and conversion | 64.6% | 26 | 249 |
| FMN-binding domain protein | 0.80 | ADU26356 | C Energy production and conversion | 14.9% | 5 | 15 |
| bifunctional acetaldehyde-CoA/alcohol dehydrogenase, ADHE | 1.32 | ADU26923 | C Energy production and conversion | 91.3% | 211 | 10944 |
| nitrogenase MoFe cofactor biosynthesis protein NifE | 1.21 | ADU27098 | C Energy production and conversion | 69.6% | 32 | 492 |
| 4Fe-4S dicluster domain-containing protein | 0.83 | ADU27685 | C Energy production and conversion | 89.3% | 2 | 4 |
| hypothetical protein | 1.25 | ADU27976 | C Energy production and conversion | 5.4% | 1 | 3 |
| P-II family nitrogen regulator | 1.41 | ADU26160 | E Amino acid transport and metabolism | 82.8% | 8 | 97 |
| ABC-type spermidine/putrescine transport system, ATPase component | 1.38 | ADU26161 | E Amino acid transport and metabolism | 66.4% | 17 | 98 |
| putrescine aminotransferase | 1.27 | ADU26164 | E Amino acid transport and metabolism | 67.3% | 29 | 476 |
| nitrogen regulatory protein P-II | 1.36 | ADU27901 | E Amino acid transport and metabolism | 88.9% | 11 | 64 |
| nitrogen regulatory protein P-II | 1.25 | ADU27902 | E Amino acid transport and metabolism | 87.3% | 11 | 57 |
| dTDP-4-amino-4,6-dideoxygalactose transaminase | 0.78 | ADU28012 | E Amino acid transport and metabolism | 6.9% | 2 | 5 |
| carbohydrate binding protein | 1.32 | ADU26013 | G Carbohydrate transport and metabolism | 1.4% | 2 | 3 |
| PTS system fructose-specific transporter subunits IIBC | 0.77 | ADU26145 | G Carbohydrate transport and metabolism | 28.7% | 12 | 74 |
| putative PTS IIA-like nitrogen-regulatory protein PtsN | 0.79 | ADU26146 | G Carbohydrate transport and metabolism | 78.4% | 9 | 35 |
| PTS fructose transporter subunit IID | 0.66 | ADU26384 | G Carbohydrate transport and metabolism | 29.0% | 9 | 206 |
| PTS sugar transporter subunit IIC | 0.67 | ADU26385 | G Carbohydrate transport and metabolism | 27.1% | 8 | 155 |
| PTS fructose transporter subunit IIB | 0.64 | ADU26386 | G Carbohydrate transport and metabolism | 88.6% | 21 | 623 |
| PTS fructose transporter subunit IIA | 0.78 | ADU26387 | G Carbohydrate transport and metabolism | 29.8% | 3 | 65 |
| 3-methyl-2-oxobutanoate hydroxymethyltransferase | 1.29 | ADU25656 | H Coenzyme transport and metabolism | 7.2% | 2 | 8 |
| 6,7-dimethyl-8-ribityllumazine synthase (Riboflavin synthase beta subunit) | 1.30 | ADU26206 | H Coenzyme transport and metabolism | 69.0% | 7 | 55 |
| 3,4-dihydroxy-2-butanone 4-phosphate synthase/GTP cyclohydrolase II | 1.25 | ADU26207 | H Coenzyme transport and metabolism | 36.8% | 13 | 47 |
| riboflavin synthase, alpha subunit | 1.34 | ADU26208 | H Coenzyme transport and metabolism | 14.2% | 3 | 6 |
| GTP 3',8-cyclase MoaA | 1.25 | ADU26419 | H Coenzyme transport and metabolism | 4.0% | 1 | 6 |
| acyl carrier protein | 0.79 | ADU28136 | I Lipid transport and metabolism | 35.5% | 3 | 61 |
| ABC-type spermidine/putrescine transport system, permease component I | 1.45 | ADU26162 | P Inorganic ion transport and metabolism | 7.6% | 2 | 7 |
| ABC-type spermidine/putrescine transport system, permease component II | 1.24 | ADU26163 | P Inorganic ion transport and metabolism | 10.2% | 5 | 10 |
| ABC-type Fe3+ transport system, periplasmic component | 0.83 | ADU27735 | P Inorganic ion transport and metabolism | 5.8% | 2 | 3 |
| ABC-type nitrate/sulfonate/bicarbonate transport system, periplasmic component | 1.28 | ADU27903 | P Inorganic ion transport and metabolism | 73.1% | 23 | 212 |
| ABC-type nitrate/sulfonate/bicarbonate transport system, ATPase component | 1.27 | ADU27905 | P Inorganic ion transport and metabolism | 36.2% | 7 | 38 |
| urea carboxylase-associated protein 2 | 1.23 | ADU27906 | P Inorganic ion transport and metabolism | 60.1% | 12 | 86 |
|  |  |  | **Poorly characterized** |  |  |  |
| Cof-like hydrolase | 0.79 | ADU26382 | S Function unknown | 3.0% | 1 | 3 |
| hypothetical protein | 1.40 | ADU26492 | S Function unknown | 8.4% | 4 | 8 |
| KH domain-containing protein | 0.73 | ADU27585 | S Function unknown | 7.8% | 1 | 8 |
| urea carboxylase-associated protein 1 | 1.47 | ADU27907 | S Function unknown | 41.9% | 5 | 29 |

**Table S2.** The differentially expressed proteins of strain YUAN-3 in 100 mM ethanol stress

| Protein name | Fold  change | Acession  number | Functional description | Coverage | Peptides | Peptide-  Spectrum  matches |
| --- | --- | --- | --- | --- | --- | --- |
|  |  |  | **No COG ID available** |  |  |  |
| hypothetical protein | 1.23 | ADU25748 | 0 | 21.2% | 4 | 26 |
| hypothetical protein | 1.22 | ADU26345 | 0 | 14.4% | 5 | 17 |
| hypothetical protein | 1.53 | ADU26774 | 0 | 1.2% | 1 | 4 |
| hypothetical protein | 1.34 | ADU27017 | 0 | 27.8% | 1 | 3 |
| hypothetical protein | 1.40 | ADU27559 | 0 | 56.3% | 5 | 37 |
| hypothetical protein | 1.20 | ADU27651 | 0 | 32.7% | 4 | 21 |
| hypothetical protein | 0.59 | ADU27745 | 0 | 14.3% | 1 | 4 |
| hypothetical protein | 1.22 | ADU27918 | 0 | 23.0% | 5 | 12 |
| hypothetical protein | 1.21 | ADU28175 | 0 | 44.1% | 5 | 72 |
| hypothetical protein | 1.21 | ADU28263 | 0 | 88.9% | 33 | 698 |
|  |  |  | **Information storage and processing** |  |  |  |
| D-tyrosyl-tRNA(Tyr) deacylase | 1.28 | ADU25926 | J Translation, ribosomal structure and biogenesis | 34.6% | 5 | 41 |
| pseudouridine synthase, RluA family | 1.76 | ADU26272 | J Translation, ribosomal structure and biogenesis | 9.3% | 2 | 3 |
| queuine tRNA-ribosyltransferase | 1.29 | ADU26959 | J Translation, ribosomal structure and biogenesis | 20.2% | 6 | 7 |
| allophanate hydrolase | 1.39 | ADU27909 | J Translation, ribosomal structure and biogenesis | 13.5% | 5 | 15 |
| TetR/AcrR family transcriptional regulator | 1.40 | ADU25620 | K Transcription | 25.4% | 6 | 22 |
| transcriptional regulator, HxlR family | 1.24 | ADU26201 | K Transcription | 20.5% | 3 | 11 |
| redox-sensing transcriptional repressor Rex | 1.25 | ADU26924 | K Transcription | 66.5% | 11 | 134 |
| RNA polymerase sigma-70 factor, sigma B/F/G subfamily | 1.20 | ADU27030 | K Transcription | 37.6% | 7 | 43 |
| Rrf2 family transcriptional regulator | 1.26 | ADU27116 | K Transcription | 53.3% | 8 | 45 |
| transcriptional regulator, MarR family | 1.21 | ADU27499 | K Transcription | 14.7% | 2 | 7 |
| transcriptional regulator, GntR family | 1.34 | ADU27755 | K Transcription | 67.6% | 18 | 178 |
| TetR/AcrR family transcriptional regulator | 1.27 | ADU27808 | K Transcription | 20.9% | 4 | 13 |
| Mor transcription activator domain protein | 0.79 | ADU27893 | K Transcription | 18.8% | 3 | 4 |
| transposase | 1.32 | ADU25645 | L Replication, recombination and repair | 9.8% | 6 | 18 |
| ATP-dependent DNA helicase RecG | 1.30 | ADU25789 | L Replication, recombination and repair | 3.5% | 3 | 3 |
| tyrosine recombinase XerC | 1.24 | ADU26950 | L Replication, recombination and repair | 35.0% | 12 | 29 |
|  |  |  | **Cellular processes and signaling** |  |  |  |
| LPXTG-motif cell wall anchor domain protein | 1.34 | ADU27225 | D Cell cycle control, cell division, chromosome partitioning | 0.3% | 1 | 4 |
| Peptidoglycan glycosyltransferase | 1.21 | ADU27573 | M Cell wall/membrane/envelope biogenesis | 5.9% | 2 | 3 |
| soluble lytic murein transglycosylase and related regulatory proteins | 1.22 | ADU28212 | M Cell wall/membrane/envelope biogenesis | 39.3% | 8 | 19 |
| Dinitrogenase iron-molybdenum cofactor biosynthesis protein | 1.25 | ADU26152 | O Posttranslational modification, protein turnover, chaperones | 95.1% | 6 | 62 |
| ATP-dependent Clp protease proteolytic subunit | 0.73 | ADU26532 | O Posttranslational modification, protein turnover, chaperones | 14.3% | 2 | 14 |
| pyruvate formate-lyase activating enzyme | 1.21 | ADU27191 | O Posttranslational modification, protein turnover, chaperones | 49.2% | 12 | 55 |
| glutathione peroxidase | 1.45 | ADU28264 | O Posttranslational modification, protein turnover, chaperones | 86.2% | 17 | 530 |
| sporulation transcriptional activator Spo0A | 1.35 | ADU26286 | T Signal transduction mechanisms | 78.2% | 19 | 218 |
| two-component sensor histidine kinase | 1.20 | ADU26679 | T Signal transduction mechanisms | 18.4% | 8 | 14 |
| diguanylate cyclase (GGDEF) domain-containing protein | 1.24 | ADU27489 | T Signal transduction mechanisms | 20.2% | 9 | 27 |
| ABC transporter permease | 0.61 | ADU25916 | V Defense mechanisms | 47.1% | 26 | 145 |
| ABC transporter ATP-binding protein | 0.54 | ADU25917 | V Defense mechanisms | 64.3% | 14 | 168 |
| ABC transporter related protein | 1.25 | ADU26168 | V Defense mechanisms | 17.4% | 5 | 8 |
| protein of unknown function DUF214 | 1.25 | ADU26169 | V Defense mechanisms | 1.9% | 2 | 4 |
| multidrug ABC transporter ATPase | 1.31 | ADU26678 | V Defense mechanisms | 3.3% | 1 | 4 |
| ABC transporter permease | 1.89 | ADU26693 | V Defense mechanisms | 12.2% | 3 | 11 |
| ABC transporter ATP-binding protein | 1.25 | ADU26986 | V Defense mechanisms | 15.5% | 5 | 13 |
| ABC-type multidrug transport system, ATPase and permease component | 1.28 | ADU27717 | V Defense mechanisms | 1.4% | 1 | 3 |
|  |  |  | **Metabolism** |  |  |  |
| putative PAS/PAC sensor protein | 1.33 | ADU25643 | C Energy production and conversion | 70.2% | 34 | 289 |
| Aldehyde Dehydrogenase | 1.32 | ADU26155 | C Energy production and conversion | 64.6% | 26 | 249 |
| FMN-binding domain protein | 0.66 | ADU26356 | C Energy production and conversion | 14.9% | 5 | 15 |
| rubredoxin | 1.28 | ADU26613 | C Energy production and conversion | 94.2% | 4 | 41 |
| Flavorubredoxin | 1.20 | ADU26614 | C Energy production and conversion | 93.0% | 57 | 1405 |
| bifunctional acetaldehyde-CoA/alcohol dehydrogenase, ADHE | 1.61 | ADU26923 | C Energy production and conversion | 91.3% | 211 | 10944 |
| nitrogenase MoFe cofactor biosynthesis protein NifE | 1.34 | ADU27098 | C Energy production and conversion | 69.6% | 32 | 492 |
| V/A-type ATP synthase subunit C | 1.22 | ADU27533 | C Energy production and conversion | 94.0% | 38 | 886 |
| V/A-type ATP synthase subunit I | 1.23 | ADU27534 | C Energy production and conversion | 42.4% | 26 | 375 |
| V/A-type ATP synthase subunit B | 1.22 | ADU27539 | C Energy production and conversion | 89.7% | 55 | 1951 |
| V/A-type ATP synthase subunit D | 1.22 | ADU27540 | C Energy production and conversion | 88.1% | 26 | 591 |
| NAD(P)-dependent alcohol dehydrogenase | 1.23 | ADU27732 | C Energy production and conversion | 72.6% | 22 | 219 |
| hydrogenase large subunit domain protein | 1.26 | ADU28188 | C Energy production and conversion | 75.7% | 39 | 820 |
| desulfoferrodoxin | 1.40 | ADU28196 | C Energy production and conversion | 95.2% | 17 | 560 |
| L-lactate dehydrogenase | 1.61 | ADU28198 | C Energy production and conversion | 79.7% | 21 | 276 |
| NADPH dehydrogenase NamA | 1.55 | ADU28199 | C Energy production and conversion | 79.2% | 19 | 130 |
| phosphate acetyltransferase | 1.23 | ADU28204 | C Energy production and conversion | 88.3% | 38 | 1037 |
| acetylornithine aminotransferase | 1.20 | ADU25731 | E Amino acid transport and metabolism | 77.8% | 27 | 458 |
| P-II family nitrogen regulator | 1.41 | ADU26160 | E Amino acid transport and metabolism | 82.8% | 8 | 97 |
| ABC-type spermidine/putrescine transport system, ATPase component | 1.51 | ADU26161 | E Amino acid transport and metabolism | 66.4% | 17 | 98 |
| putrescine aminotransferase | 1.45 | ADU26164 | E Amino acid transport and metabolism | 67.3% | 29 | 476 |
| aspartate aminotransferase family protein | 0.80 | ADU26725 | E Amino acid transport and metabolism | 87.2% | 33 | 560 |
| beta-alanine synthase | 0.83 | ADU26726 | E Amino acid transport and metabolism | 91.1% | 35 | 553 |
| phosphoribosyl-ATP pyrophosphatase HisE | 1.40 | ADU26970 | E Amino acid transport and metabolism | 50.0% | 5 | 32 |
| Phosphoribosyl-AMP cyclohydrolase HisI1 | 1.36 | ADU26971 | E Amino acid transport and metabolism | 73.1% | 7 | 87 |
| imidazole glycerol phosphate synthase subunit HisF | 1.28 | ADU26972 | E Amino acid transport and metabolism | 86.9% | 20 | 297 |
| 1-(5-phosphoribosyl)-5-[(5-phosphoribosylamino)methylideneamino] imidazole-4-carboxamide isomerase HisA | 1.24 | ADU26973 | E Amino acid transport and metabolism | 84.7% | 20 | 216 |
| imidazoleglycerol-phosphate dehydratase HisB | 1.21 | ADU26975 | E Amino acid transport and metabolism | 45.8% | 8 | 66 |
| histidinol dehydrogenase HisD | 1.23 | ADU26977 | E Amino acid transport and metabolism | 65.2% | 34 | 612 |
| ATP phosphoribosyltransferase HisG | 1.31 | ADU26978 | E Amino acid transport and metabolism | 72.0% | 13 | 262 |
| ATP phosphoribosyltransferase regulatory subunit HisZ | 1.31 | ADU26979 | E Amino acid transport and metabolism | 89.4% | 34 | 660 |
| hypothetical protein | 1.24 | ADU27095 | E Amino acid transport and metabolism | 12.6% | 5 | 9 |
| 5-methyltetrahydropteroyltriglutamate--homocysteine S-methyltransferase | 1.25 | ADU27657 | E Amino acid transport and metabolism | 77.1% | 51 | 406 |
| threonine dehydratase | 1.23 | ADU27756 | E Amino acid transport and metabolism | 40.4% | 12 | 149 |
| histidinol-phosphate transaminase | 1.20 | ADU27760 | E Amino acid transport and metabolism | 69.6% | 27 | 535 |
| ABC-type branched-chain amino acid transport system, ATPase component LivF | 0.72 | ADU27844 | E Amino acid transport and metabolism | 78.8% | 19 | 142 |
| ABC-type branched-chain amino acid transport system, ATPase component livG | 0.72 | ADU27845 | E Amino acid transport and metabolism | 60.7% | 15 | 165 |
| ABC-type branched-chain amino acid transport system, permease component LivM | 0.62 | ADU27846 | E Amino acid transport and metabolism | 15.8% | 6 | 26 |
| ABC-type branched-chain amino acid transport system, periplasmic component LivK | 0.69 | ADU27848 | E Amino acid transport and metabolism | 89.1% | 36 | 543 |
| nitrogen regulatory protein P-II | 2.04 | ADU27901 | E Amino acid transport and metabolism | 88.9% | 11 | 64 |
| nitrogen regulatory protein P-II | 1.59 | ADU27902 | E Amino acid transport and metabolism | 87.3% | 11 | 57 |
| urea carboxylase | 1.50 | ADU27908 | E Amino acid transport and metabolism | 23.0% | 25 | 66 |
| cysteine desulfurase, SufS subfamily | 1.21 | ADU28120 | E Amino acid transport and metabolism | 83.9% | 26 | 522 |
| amino acid-binding ACT domain protein | 1.22 | ADU28260 | E Amino acid transport and metabolism | 76.2% | 8 | 40 |
| Adenine deaminase | 1.22 | ADU26402 | F Nucleotide transport and metabolism | 74.3% | 33 | 184 |
| rRNA maturation RNase YbeY | 1.21 | ADU26740 | F Nucleotide transport and metabolism | 59.8% | 7 | 84 |
| carbohydrate binding protein | 0.75 | ADU26013 | G Carbohydrate transport and metabolism | 1.4% | 2 | 3 |
| PTS system fructose-specific transporter subunits IIBC | 0.78 | ADU26145 | G Carbohydrate transport and metabolism | 28.7% | 12 | 74 |
| putative PTS IIA-like nitrogen-regulatory protein PtsN | 0.73 | ADU26146 | G Carbohydrate transport and metabolism | 78.4% | 9 | 35 |
| carbohydrate ABC transporter substrate-binding protein | 1.24 | ADU26315 | G Carbohydrate transport and metabolism | 88.2% | 33 | 556 |
| beta-galactosidase | 1.21 | ADU26457 | G Carbohydrate transport and metabolism | 8.8% | 3 | 4 |
| 2,3-diphosphoglycerate-dependent phosphoglycerate mutase | 1.20 | ADU26920 | G Carbohydrate transport and metabolism | 80.2% | 23 | 435 |
| Phosphoglycerate kinase | 1.28 | ADU27083 | G Carbohydrate transport and metabolism | 96.2% | 79 | 4570 |
| triosephosphate isomerase | 1.23 | ADU27084 | G Carbohydrate transport and metabolism | 94.4% | 50 | 1532 |
| 2,3-bisphosphoglycerate-independent phosphoglycerate mutase | 1.32 | ADU27085 | G Carbohydrate transport and metabolism | 97.2% | 69 | 2090 |
| PTS system fructose-specific EIIABC component | 1.47 | ADU27519 | G Carbohydrate transport and metabolism | 45.9% | 28 | 148 |
| Phosphotransferase system, phosphocarrier protein HPr | 1.25 | ADU27581 | G Carbohydrate transport and metabolism | 82.8% | 8 | 129 |
| glyceraldehyde-3-phosphate dehydrogenase, type I | 1.23 | ADU28097 | G Carbohydrate transport and metabolism | 92.1% | 102 | 7526 |
| PTS mannitol transporter subunit IICB | 1.34 | ADU28246 | G Carbohydrate transport and metabolism | 3.4% | 2 | 6 |
| 3-methyl-2-oxobutanoate hydroxymethyltransferase | 1.30 | ADU25656 | H Coenzyme transport and metabolism | 7.2% | 2 | 8 |
| pyridoxal 5'-phosphate synthase lyase subunit PdxS | 1.23 | ADU25721 | H Coenzyme transport and metabolism | 67.5% | 17 | 131 |
| GTP 3',8-cyclase MoaA | 0.71 | ADU26419 | H Coenzyme transport and metabolism | 4.0% | 1 | 6 |
| nicotinate phosphoribosyltransferase | 1.24 | ADU26597 | H Coenzyme transport and metabolism | 70.7% | 35 | 278 |
| thiamine biosynthesis lipoprotein ApbE | 1.23 | ADU27769 | H Coenzyme transport and metabolism | 14.8% | 4 | 12 |
| acyl-CoA hydrolase | 1.29 | ADU28191 | I Lipid transport and metabolism | 64.1% | 14 | 166 |
| CoA-disulfide reductase | 1.42 | ADU25715 | P Inorganic ion transport and metabolism | 54.0% | 39 | 179 |
| ABC-type spermidine/putrescine transport system, permease component I | 1.51 | ADU26162 | P Inorganic ion transport and metabolism | 7.6% | 2 | 7 |
| ABC-type spermidine/putrescine transport system, permease component II | 1.43 | ADU26163 | P Inorganic ion transport and metabolism | 10.2% | 5 | 10 |
| methyl-accepting chemotaxis protein | 1.29 | ADU26469 | P Inorganic ion transport and metabolism | 5.1% | 4 | 15 |
| arsenical-resistance protein | 1.28 | ADU26530 | P Inorganic ion transport and metabolism | 9.4% | 3 | 5 |
| copper chaperone | 1.53 | ADU26619 | P Inorganic ion transport and metabolism | 58.0% | 4 | 14 |
| copper-translocating P-type ATPase | 1.23 | ADU26620 | P Inorganic ion transport and metabolism | 41.3% | 29 | 112 |
| Ferrous iron transport protein A | 1.22 | ADU26659 | P Inorganic ion transport and metabolism | 90.4% | 8 | 113 |
| ABC-type nitrate/sulfonate/bicarbonate transport system, periplasmic component | 0.79 | ADU26719 | P Inorganic ion transport and metabolism | 80.4% | 25 | 640 |
| cobalt/nickel ABC transporter ATP-binding protein | 1.22 | ADU26833 | P Inorganic ion transport and metabolism | 24.3% | 4 | 7 |
| ABC-type nitrate/sulfonate/bicarbonate transport system, periplasmic component | 1.92 | ADU27903 | P Inorganic ion transport and metabolism | 73.1% | 23 | 212 |
| ABC-type nitrate/sulfonate/bicarbonate transport system, ATPase component | 1.66 | ADU27905 | P Inorganic ion transport and metabolism | 36.2% | 7 | 38 |
| urea carboxylase-associated protein 2 | 1.92 | ADU27906 | P Inorganic ion transport and metabolism | 60.1% | 12 | 86 |
| heavy metal translocating P-type ATPase | 1.24 | ADU28218 | P Inorganic ion transport and metabolism | 16.0% | 11 | 29 |
|  |  |  | **Poorly characterized** |  |  |  |
| DUF815 domain-containing protein | 1.21 | ADU25767 | S Function unknown | 79.8% | 29 | 227 |
| hypothetical protein | 1.35 | ADU25811 | S Function unknown | 51.4% | 10 | 67 |
| hypothetical protein | 1.20 | ADU25930 | S Function unknown | 74.6% | 10 | 123 |
| hypothetical protein | 1.23 | ADU25944 | S Function unknown | 36.6% | 10 | 48 |
| hypothetical protein | 1.21 | ADU26039 | S Function unknown | 6.1% | 1 | 15 |
| hypothetical protein | 1.28 | ADU26186 | S Function unknown | 11.2% | 2 | 4 |
| hypothetical protein | 1.25 | ADU26284 | S Function unknown | 73.6% | 9 | 47 |
| hypothetical protein | 1.31 | ADU26362 | S Function unknown | 73.3% | 11 | 112 |
| hypothetical protein | 1.48 | ADU26492 | S Function unknown | 8.4% | 4 | 8 |
| metallophosphoesterase | 1.28 | ADU26649 | S Function unknown | 13.5% | 4 | 15 |
| SpoIIIAH-like family protein | 1.27 | ADU26757 | S Function unknown | 5.9% | 1 | 6 |
| Protein of unknown function DUF1998 | 1.21 | ADU26810 | S Function unknown | 6.1% | 4 | 7 |
| hypothetical protein | 1.35 | ADU26849 | S Function unknown | 85.3% | 11 | 73 |
| DNA mismatch repair protein MutS | 1.21 | ADU27015 | S Function unknown | 32.9% | 2 | 3 |
| hypothetical protein | 1.33 | ADU27125 | S Function unknown | 6.0% | 3 | 3 |
| nitroreductase | 1.35 | ADU27149 | S Function unknown | 83.9% | 16 | 175 |
| hypothetical protein | 1.20 | ADU27481 | S Function unknown | 83.7% | 7 | 40 |
| hypothetical protein | 1.29 | ADU27532 | S Function unknown | 78.6% | 10 | 84 |
| V/A-type ATP synthase subunit E | 1.23 | ADU27537 | S Function unknown | 89.4% | 25 | 616 |
| hypothetical protein | 1.32 | ADU27553 | S Function unknown | 10.9% | 2 | 6 |
| hypothetical protein | 1.57 | ADU27666 | S Function unknown | 17.6% | 2 | 5 |
| hypothetical protein | 0.79 | ADU27743 | S Function unknown | 80.0% | 7 | 231 |
| carbon-nitrogen hydrolase family protein | 0.68 | ADU27842 | S Function unknown | 75.4% | 21 | 660 |
| cytidylate kinase-like family protein | 0.75 | ADU27843 | S Function unknown | 70.0% | 14 | 113 |
| hypothetical protein | 1.22 | ADU27868 | S Function unknown | 5.0% | 8 | 17 |
| urea carboxylase-associated protein 1 | 2.12 | ADU27907 | S Function unknown | 41.9% | 5 | 29 |
| hypothetical protein | 1.31 | ADU28111 | S Function unknown | 3.6% | 1 | 3 |
| DUF951 domain-containing protein | 1.22 | ADU28208 | S Function unknown | 26.2% | 2 | 5 |
| tRNA-specific adenosine deaminase | 1.24 | ADU27089 | Nucleotide transport and metabolism / Translation, ribosomal structure and biogenesis | 34.4% | 5 | 17 |

**Table S3.** The differentially expressed proteins of strain YUAN-3 in 200 mM ethanol stress

| Protein name | Fold  change | Acession  number | Functional description | Coverage | Peptides | Peptide-  spectrum matches |
| --- | --- | --- | --- | --- | --- | --- |
|  |  |  | **No COG ID available** |  |  |  |
| hypothetical protein | 0.80 | ADU25942 | 0 | 8.8% | 3 | 7 |
| hypothetical protein | 0.82 | ADU26507 | 0 | 40.7% | 9 | 35 |
| hypothetical protein | 1.33 | ADU27017 | 0 | 27.8% | 1 | 3 |
| hypothetical protein | 0.48 | ADU27745 | 0 | 14.3% | 1 | 4 |
| flagellar motor switch protein FliG | 1.29 | ADU28070 | 0 | 10.2% | 3 | 10 |
| hypothetical protein | 1.40 | ADU28175 | 0 | 44.1% | 5 | 72 |
| hypothetical protein | 0.74 | ADU28268 | 0 | 68.9% | 6 | 33 |
|  |  |  | **Information storage and processing** |  |  |  |
| pseudouridine synthase, RluA family | 0.82 | ADU26272 | J Translation, ribosomal structure and biogenesis | 9.3% | 2 | 3 |
| ribosomal protein L7/L12 | 0.76 | ADU27595 | J Translation, ribosomal structure and biogenesis | 77.8% | 13 | 368 |
| RNA binding S1 domain protein | 0.56 | ADU28206 | J Translation, ribosomal structure and biogenesis | 29.7% | 9 | 18 |
| transcriptional regulator, MarR family | 0.81 | ADU25602 | K Transcription | 39.7% | 6 | 17 |
| TetR/AcrR family transcriptional regulator | 1.31 | ADU25620 | K Transcription | 25.4% | 6 | 22 |
| ATP-dependent transcriptional regulator, MalT-like, LuxR family | 0.79 | ADU25992 | K Transcription | 1.6% | 1 | 3 |
| transcriptional regulator, GntR family | 1.22 | ADU26287 | K Transcription | 35.8% | 7 | 15 |
| transcriptional antiterminator, BglG | 0.81 | ADU26290 | K Transcription | 64.0% | 16 | 110 |
| Rrf2 family transcriptional regulator | 0.81 | ADU26612 | K Transcription | 33.3% | 5 | 21 |
| RNA polymerase sigma-70 factor, sigma B/F/G subfamily | 1.32 | ADU27030 | K Transcription | 37.6% | 7 | 43 |
| Mor transcription activator domain protein | 0.69 | ADU27893 | K Transcription | 18.8% | 3 | 4 |
| TetR/AcrR family transcriptional regulator | 0.68 | ADU28238 | K Transcription | 81.3% | 17 | 139 |
| transcriptional regulator, XRE family | 0.82 | ADU26048 | L Replication, recombination and repair | 19.1% | 8 | 29 |
| crossover junction endodeoxyribonuclease RuvC | 0.82 | ADU26581 | L Replication, recombination and repair | 19.8% | 3 | 4 |
| DNA primase | 0.82 | ADU26610 | L Replication, recombination and repair | 6.7% | 4 | 4 |
| tyrosine recombinase XerD | 0.77 | ADU26951 | L Replication, recombination and repair | 20.4% | 5 | 18 |
|  |  |  | **Cellular processes and signaling** |  |  |  |
| D-alanyl-D-alanine carboxypeptidase | 0.48 | ADU26699 | M Cell wall/membrane/envelope biogenesis | 11.9% | 5 | 15 |
| DUF3794 domain-containing protein | 0.54 | ADU27270 | M Cell wall/membrane/envelope biogenesis | 53.1% | 23 | 107 |
| Peptidoglycan glycosyltransferase | 1.22 | ADU27573 | M Cell wall/membrane/envelope biogenesis | 5.9% | 2 | 3 |
| murein L,D-transpeptidase | 0.79 | ADU27788 | M Cell wall/membrane/envelope biogenesis | 23.2% | 4 | 17 |
| glycosyl transferase family 2 | 0.81 | ADU28040 | M Cell wall/membrane/envelope biogenesis | 60.7% | 38 | 361 |
| LrgB family protein | 0.78 | ADU28186 | M Cell wall/membrane/envelope biogenesis | 7.7% | 1 | 5 |
| cell surface antigen BspA-like protein | 0.76 | ADU26128 | N Cell motility | 10.4% | 7 | 15 |
| flagellar motor protein MotA | 0.82 | ADU28031 | N Cell motility | 55.3% | 14 | 56 |
| flagellar export chaperone FliS | 0.80 | ADU28033 | N Cell motility | 47.6% | 9 | 44 |
| flagellin | 0.71 | ADU28041 | N Cell motility | 37.5% | 19 | 370 |
| flagellar motor protein MotP | 0.75 | ADU28061 | N Cell motility | 13.9% | 4 | 8 |
| flagellar hook-basal body protein | 0.70 | ADU28063 | N Cell motility | 30.4% | 7 | 54 |
| ATP-dependent Clp protease proteolytic subunit | 0.62 | ADU26532 | O Posttranslational modification, protein turnover, chaperones | 14.3% | 2 | 14 |
| anaerobic ribonucleoside-triphosphate reductase activating protein | 0.79 | ADU27988 | O Posttranslational modification, protein turnover, chaperones | 24.9% | 5 | 26 |
| multi-sensor signal transduction histidine kinase | 0.74 | ADU25837 | T Signal transduction mechanisms | 29.6% | 12 | 49 |
| sporulation transcriptional activator Spo0A | 1.51 | ADU26286 | T Signal transduction mechanisms | 78.2% | 19 | 218 |
| serine protein kinase, PrkA | 0.61 | ADU26297 | T Signal transduction mechanisms | 40.5% | 24 | 58 |
| anti-sigma F factor | 1.32 | ADU27029 | T Signal transduction mechanisms | 90.4% | 17 | 261 |
| diguanylate cyclase (GGDEF) domain-containing protein | 0.82 | ADU27150 | T Signal transduction mechanisms | 6.3% | 3 | 4 |
| carbon storage regulator, CsrA | 0.81 | ADU28042 | T Signal transduction mechanisms | 28.2% | 3 | 9 |
| stage II sporulation protein E | 1.28 | ADU28240 | T Signal transduction mechanisms | 15.3% | 12 | 33 |
| ABC transporter permease | 0.58 | ADU25916 | V Defense mechanisms | 47.1% | 26 | 145 |
| ABC transporter ATP-binding protein | 0.57 | ADU25917 | V Defense mechanisms | 64.3% | 14 | 168 |
| ABC transporter related protein | 0.82 | ADU26002 | V Defense mechanisms | 57.2% | 32 | 492 |
| ABC transporter related protein | 1.56 | ADU26168 | V Defense mechanisms | 17.4% | 5 | 8 |
| protein of unknown function DUF214 | 1.68 | ADU26169 | V Defense mechanisms | 1.9% | 2 | 4 |
| ABC-type lipoprotein export system, ATPase component | 1.72 | ADU26590 | V Defense mechanisms | 92.6% | 21 | 400 |
| ABC-type lipoprotein export system, permease component | 1.57 | ADU26591 | V Defense mechanisms | 51.0% | 34 | 392 |
| ABC transporter permease | 1.54 | ADU26693 | V Defense mechanisms | 12.2% | 3 | 11 |
| ABC-type multidrug transport system, ATPase and permease component | 1.29 | ADU27717 | V Defense mechanisms | 1.4% | 1 | 3 |
|  |  |  | **Metabolism** |  |  |  |
| putative PAS/PAC sensor protein | 1.23 | ADU25643 | C Energy production and conversion | 70.2% | 34 | 289 |
| iron-containing alcohol dehydrogenase | 0.75 | ADU26157 | C Energy production and conversion | 16.2% | 5 | 13 |
| citrate/2-methylcitrate synthase | 1.28 | ADU26344 | C Energy production and conversion | 85.8% | 43 | 635 |
| rubredoxin | 1.25 | ADU26613 | C Energy production and conversion | 94.2% | 4 | 41 |
| iron-containing alcohol dehydrogenase | 0.82 | ADU26715 | C Energy production and conversion | 69.4% | 26 | 391 |
| bifunctional acetaldehyde-CoA/alcohol dehydrogenase, ADHE | 1.52 | ADU26923 | C Energy production and conversion | 91.3% | 211 | 10944 |
| aconitate hydratase A | 1.21 | ADU26926 | C Energy production and conversion | 86.0% | 61 | 1510 |
| ferredoxin, 2Fe-2S | 1.45 | ADU27096 | C Energy production and conversion | 54.4% | 4 | 95 |
| nitrogenase MoFe cofactor biosynthesis protein NifB | 1.24 | ADU27097 | C Energy production and conversion | 89.0% | 78 | 1215 |
| molybdenum-dependent nitrogenase (Mo-nitrogenase) molybdenum-iron protein beta chain ( Mo-nitrogenase component I subunit beta ) | 1.21 | ADU27099 | C Energy production and conversion | 90.7% | 60 | 1552 |
| molybdenum-dependent nitrogenase (Mo-nitrogenase) molybdenum-iron protein alpha chain ( Mo-nitrogenase component I subunit alpha ) | 1.23 | ADU27100 | C Energy production and conversion | 92.1% | 69 | 2294 |
| vanadium-dependent nitrogenase (V-nitrogenase) VFe cofactor biosynthesis protein VnfN | 0.74 | ADU27247 | C Energy production and conversion | 20.8% | 8 | 17 |
| 1,3-propanediol dehydrogenase | 0.68 | ADU27753 | C Energy production and conversion | 81.7% | 29 | 647 |
| hypothetical protein | 1.21 | ADU27976 | C Energy production and conversion | 5.4% | 1 | 3 |
| hydrogenase large subunit domain protein | 1.21 | ADU28188 | C Energy production and conversion | 75.7% | 39 | 820 |
| desulfoferrodoxin | 1.30 | ADU28196 | C Energy production and conversion | 95.2% | 17 | 560 |
| argininosuccinate synthase | 1.22 | ADU25737 | E Amino acid transport and metabolism | 70.8% | 29 | 531 |
| amino acid/polyamine/organocation transporter, APC superfamily | 0.81 | ADU25761 | E Amino acid transport and metabolism | 31.8% | 20 | 228 |
| P-II family nitrogen regulator | 0.72 | ADU26160 | E Amino acid transport and metabolism | 82.8% | 8 | 97 |
| ABC-type spermidine/putrescine transport system, ATPase component | 0.80 | ADU26161 | E Amino acid transport and metabolism | 66.4% | 17 | 98 |
| dihydropyrimidine dehydrogenase subunit A | 0.79 | ADU26723 | E Amino acid transport and metabolism | 38.7% | 7 | 29 |
| aspartate aminotransferase family protein | 0.70 | ADU26725 | E Amino acid transport and metabolism | 87.2% | 33 | 560 |
| beta-alanine synthase | 0.77 | ADU26726 | E Amino acid transport and metabolism | 91.1% | 35 | 553 |
| imidazole glycerol phosphate synthase subunit HisF | 1.21 | ADU26972 | E Amino acid transport and metabolism | 86.9% | 20 | 297 |
| ATP phosphoribosyltransferase HisG | 1.26 | ADU26978 | E Amino acid transport and metabolism | 72.0% | 13 | 262 |
| ATP phosphoribosyltransferase regulatory subunit HisZ | 1.22 | ADU26979 | E Amino acid transport and metabolism | 89.4% | 34 | 660 |
| hypothetical protein | 1.21 | ADU27095 | E Amino acid transport and metabolism | 12.6% | 5 | 9 |
| nitrogen regulatory protein P-II | 1.26 | ADU27102 | E Amino acid transport and metabolism | 76.9% | 9 | 379 |
| cystathionine gamma-synthase | 0.60 | ADU27251 | E Amino acid transport and metabolism | 13.4% | 3 | 10 |
| Aspartate/methionine/tyrosine aminotransferase | 0.73 | ADU27252 | E Amino acid transport and metabolism | 11.0% | 6 | 8 |
| Aspartate/methionine/tyrosine aminotransferase | 0.67 | ADU27256 | E Amino acid transport and metabolism | 5.4% | 2 | 3 |
| histidinol-phosphate transaminase | 0.67 | ADU27760 | E Amino acid transport and metabolism | 69.6% | 27 | 535 |
| arginine deiminase | 0.83 | ADU27762 | E Amino acid transport and metabolism | 94.0% | 35 | 1119 |
| 4-aminobutyrate transaminase | 0.83 | ADU27764 | E Amino acid transport and metabolism | 90.0% | 42 | 1137 |
| D-3-phosphoglycerate dehydrogenase (PHGDH) | 0.79 | ADU27765 | E Amino acid transport and metabolism | 76.5% | 21 | 291 |
| ABC-type branched-chain amino acid transport system, ATPase component LivF | 0.75 | ADU27844 | E Amino acid transport and metabolism | 78.8% | 19 | 142 |
| ABC-type branched-chain amino acid transport system, ATPase component livG | 0.77 | ADU27845 | E Amino acid transport and metabolism | 60.7% | 15 | 165 |
| ABC-type branched-chain amino acid transport system, permease component LivM | 0.63 | ADU27846 | E Amino acid transport and metabolism | 15.8% | 6 | 26 |
| ABC-type branched-chain amino acid transport system, periplasmic component LivK | 0.70 | ADU27848 | E Amino acid transport and metabolism | 89.1% | 36 | 543 |
| nitrogen regulatory protein P-II | 0.82 | ADU27901 | E Amino acid transport and metabolism | 88.9% | 11 | 64 |
| nitrogen regulatory protein P-II | 0.82 | ADU27902 | E Amino acid transport and metabolism | 87.3% | 11 | 57 |
| 5'-deoxynucleotidase | 0.63 | ADU28254 | F Nucleotide transport and metabolism | 36.3% | 6 | 25 |
| carbohydrate binding protein | 0.59 | ADU26013 | G Carbohydrate transport and metabolism | 1.4% | 2 | 3 |
| mannose-6-phosphate isomerase, class I | 0.82 | ADU26143 | G Carbohydrate transport and metabolism | 76.5% | 17 | 81 |
| PTS system fructose-specific transporter subunits IIBC | 0.74 | ADU26145 | G Carbohydrate transport and metabolism | 28.7% | 12 | 74 |
| putative PTS IIA-like nitrogen-regulatory protein PtsN | 0.66 | ADU26146 | G Carbohydrate transport and metabolism | 78.4% | 9 | 35 |
| ROK family protein | 1.28 | ADU26311 | G Carbohydrate transport and metabolism | 33.8% | 7 | 20 |
| carbohydrate ABC transporter substrate-binding protein | 1.80 | ADU26315 | G Carbohydrate transport and metabolism | 88.2% | 33 | 556 |
| sugar ABC transporter substrate-binding protein | 0.82 | ADU26392 | G Carbohydrate transport and metabolism | 17.3% | 5 | 13 |
| PTS sugar transporter subunit IIC | 0.82 | ADU26412 | G Carbohydrate transport and metabolism | 6.3% | 5 | 15 |
| Mannosyl-glycoprotein endo-beta-N-acetylglucosaminidase | 1.21 | ADU26966 | G Carbohydrate transport and metabolism | 45.6% | 28 | 411 |
| glyceraldehyde-3-phosphate dehydrogenase, type I | 0.71 | ADU27040 | G Carbohydrate transport and metabolism | 47.0% | 13 | 58 |
| PTS system fructose-specific EIIABC component | 1.77 | ADU27519 | G Carbohydrate transport and metabolism | 45.9% | 28 | 148 |
| Spore germination protein YaaH | 0.66 | ADU28166 | G Carbohydrate transport and metabolism | 31.0% | 9 | 22 |
| pyridoxal 5'-phosphate synthase lyase subunit PdxS | 0.74 | ADU25721 | H Coenzyme transport and metabolism | 67.5% | 17 | 131 |
| 6,7-dimethyl-8-ribityllumazine synthase (Riboflavin synthase beta subunit) | 0.73 | ADU26206 | H Coenzyme transport and metabolism | 69.0% | 7 | 55 |
| 3,4-dihydroxy-2-butanone 4-phosphate synthase/GTP cyclohydrolase II | 0.68 | ADU26207 | H Coenzyme transport and metabolism | 36.8% | 13 | 47 |
| riboflavin synthase, alpha subunit | 0.80 | ADU26208 | H Coenzyme transport and metabolism | 14.2% | 3 | 6 |
| GTP 3',8-cyclase MoaA | 0.54 | ADU26419 | H Coenzyme transport and metabolism | 4.0% | 1 | 6 |
| molybdenum-pterin-binding protein | 1.20 | ADU27105 | H Coenzyme transport and metabolism | 91.2% | 7 | 245 |
| 2C-methyl-D-erythritol 2,4-cyclodiphosphate synthase | 0.81 | ADU26871 | I Lipid transport and metabolism | 29.3% | 4 | 7 |
| drug resistance transporter, EmrB/QacA subfamily | 0.79 | ADU25603 | P Inorganic ion transport and metabolism | 4.7% | 3 | 8 |
| ABC-type spermidine/putrescine transport system, permease component I | 0.78 | ADU26162 | P Inorganic ion transport and metabolism | 7.6% | 2 | 7 |
| carbohydrate ABC transporter permease | 1.36 | ADU26313 | P Inorganic ion transport and metabolism | 13.5% | 5 | 18 |
| magnesium-transporting ATPase | 0.80 | ADU26681 | P Inorganic ion transport and metabolism | 5.1% | 4 | 5 |
| ABC-type nitrate/sulfonate/bicarbonate transport system, ATPase component | 0.83 | ADU26716 | P Inorganic ion transport and metabolism | 81.6% | 18 | 284 |
| ABC-type nitrate/sulfonate/bicarbonate transport system, permease component | 0.81 | ADU26717 | P Inorganic ion transport and metabolism | 24.0% | 5 | 20 |
| ABC-type nitrate/sulfonate/bicarbonate transport system, permease component | 0.81 | ADU26718 | P Inorganic ion transport and metabolism | 9.7% | 2 | 8 |
| ABC-type nitrate/sulfonate/bicarbonate transport system, periplasmic component | 0.80 | ADU26719 | P Inorganic ion transport and metabolism | 80.4% | 25 | 640 |
| nitrogenase iron protein (nitrogenase component II) | 1.23 | ADU27103 | P Inorganic ion transport and metabolism | 75.9% | 41 | 2437 |
| methionine ABC transporter substrate-binding protein | 0.73 | ADU27257 | P Inorganic ion transport and metabolism | 6.5% | 2 | 3 |
| polyketide synthase | 0.73 | ADU25856 | Q Secondary metabolites biosynthesis, transport and catabolism | 2.7% | 2 | 3 |
| cysteine hydrolase | 1.36 | ADU26566 | Q Secondary metabolites biosynthesis, transport and catabolism | 50.8% | 8 | 38 |
|  |  |  | **Poorly characterized** |  |  |  |
| lipase class 3 | 0.80 | ADU25993 | S Function unknown | 12.2% | 3 | 7 |
| Radical SAM domain protein | 0.79 | ADU25998 | S Function unknown | 89.3% | 47 | 1138 |
| 3-oxoacyl-ACP reductase/3-oxoacyl-[acyl-carrier protein] reductase | 1.27 | ADU26251 | S Function unknown | 72.5% | 17 | 381 |
| hypothetical protein | 1.42 | ADU26492 | S Function unknown | 8.4% | 4 | 8 |
| TetR/AcrR family transcriptional regulator | 0.78 | ADU26586 | S Function unknown | 27.5% | 3 | 3 |
| protein of unknown function DUF1292 | 1.23 | ADU26664 | S Function unknown | 50.9% | 3 | 31 |
| SpoIIIAH-like family protein | 0.77 | ADU26757 | S Function unknown | 5.9% | 1 | 6 |
| hypothetical protein | 1.30 | ADU26849 | S Function unknown | 85.3% | 11 | 73 |
| flavodoxin | 0.66 | ADU27041 | S Function unknown | 38.3% | 6 | 34 |
| YitT family protein | 0.82 | ADU27264 | S Function unknown | 10.9% | 2 | 5 |
| stage IV sporulation protein A | 0.48 | ADU27269 | S Function unknown | 63.0% | 26 | 158 |
| KH domain-containing protein | 0.72 | ADU27585 | S Function unknown | 7.8% | 1 | 8 |
| hypothetical protein | 0.77 | ADU27666 | S Function unknown | 17.6% | 2 | 5 |
| hypothetical protein | 0.78 | ADU27743 | S Function unknown | 80.0% | 7 | 231 |
| carbon-nitrogen hydrolase family protein | 0.73 | ADU27842 | S Function unknown | 75.4% | 21 | 660 |
| cytidylate kinase-like family protein | 0.75 | ADU27843 | S Function unknown | 70.0% | 14 | 113 |
| Predicted Fe2+/Mn2+ transporter | 0.81 | ADU28214 | S Function unknown | 15.9% | 5 | 24 |
| YhgE/Pip C-terminal domain protein | 0.66 | ADU28239 | S Function unknown | 28.4% | 26 | 221 |
| thioredoxin family protein | 0.74 | ADU27542 | Energy production and conversion / Posttranslational modification, protein turnover, chaperones | 14.8% | 2 | 5 |
| Prepilin peptidase | 0.82 | ADU26359 | N Cell motility / Posttranslational modification, protein turnover, chaperones / Intracellular trafficking, secretion, and vesicular transport | 13.1% | 3 | 4 |
| flagellar protein export ATPase FliI | 0.81 | ADU28068 | N Cell motility / Intracellular trafficking, secretion, and vesicular transport | 37.6% | 15 | 37 |

**Table S4.** KEGG pathway enrichment of the differentially expressed proteins in strain YUAN-3

| Term | ID | Input number | Background number | P-Value |
| --- | --- | --- | --- | --- |
| **Cluster 1** |  |  |  |  |
| ABC transporters | eha02010 | 5 | 66 | 8.85E-04 |
| Phosphotransferase system (PTS) | eha02060 | 2 | 17 | 1.78E-02 |
| Fructose and mannose metabolism | eha00051 | 2 | 23 | 2.99E-02 |
| Cysteine and methionine metabolism | eha00270 | 2 | 24 | 3.22E-02 |
| **Cluster 2** |  |  |  |  |
| Riboflavin metabolism | eha00740 | 3 | 5 | 7.51E-06 |
| Sulfur relay system | eha04122 | 1 | 6 | 3.81E-02 |
| Folate biosynthesis | eha00790 | 1 | 6 | 3.81E-02 |
| **Cluster 3** |  |  |  |  |
| Oxidative phosphorylation | eha00190 | 5 | 14 | 5.14E-05 |
| Two-component system | eha02020 | 5 | 32 | 1.40E-03 |
| Flagellar assembly | eha02040 | 4 | 28 | 5.77E-03 |
| **Cluster 4** |  |  |  |  |
| Phosphotransferase system (PTS) | eha02060 | 3 | 17 | 9.35E-05 |
| Fructose and mannose metabolism | eha00051 | 2 | 23 | 5.97E-03 |
| Amino sugar and nucleotide sugar metabolism | eha00520 | 2 | 37 | 1.42E-02 |
| **Cluster 5** |  |  |  |  |
| Histidine metabolism | eha00340 | 8 | 13 | 4.00E-07 |
| Biosynthesis of amino acids | eha01230 | 16 | 109 | 1.00E-05 |
| Biosynthesis of secondary metabolites | eha01110 | 19 | 179 | 9.14E-05 |
| Glycolysis / Gluconeogenesis | eha00010 | 7 | 31 | 4.07E-04 |
| Metabolic pathways | eha01100 | 27 | 388 | 1.99E-03 |
| Microbial metabolism in diverse environments | eha01120 | 10 | 100 | 7.15E-03 |
| Arginine biosynthesis | eha00220 | 3 | 14 | 2.36E-02 |
| Atrazine degradation | eha00791 | 2 | 5 | 2.58E-02 |
| Carbon metabolism | eha01200 | 6 | 58 | 3.07E-02 |
| Methane metabolism | eha00680 | 3 | 16 | 3.19E-02 |
| Glycine, serine and threonine metabolism | eha00260 | 3 | 16 | 3.19E-02 |
| Selenocompound metabolism | eha00450 | 2 | 6 | 3.35E-02 |
| **Cluster 6** |  |  |  |  |
| Microbial metabolism in diverse environments | eha01120 | 7 | 100 | 3.75E-04 |
| Chloroalkane and chloroalkene degradation | eha00625 | 3 | 10 | 5.46E-04 |
| Nitrogen metabolism | eha00910 | 3 | 16 | 1.75E-03 |
| Glyoxylate and dicarboxylate metabolism | eha00630 | 2 | 10 | 1.02E-02 |
| Citrate cycle | eha00020 | 2 | 11 | 1.20E-02 |
| 2-Oxocarboxylic acid metabolism | eha01210 | 2 | 22 | 3.87E-02 |
| Carbon metabolism | eha01200 | 3 | 58 | 4.47E-02 |

**Table S5.** The ten most numerous peptide-spectrum matches of all the identified proteins in strain YUAN-3

| Acession number | Protein name | Coverage peptide length | Coverage | Peptide-spectrum matches |
| --- | --- | --- | --- | --- |
| ADU25947 | chaperonin GroEL | 499 | 91.22% | 10984 |
| **ADU26923** | **bifunctional acetaldehyde-CoA/alcohol dehydrogenase (ADHE)** | **795** | **91.27%** | **10944** |
| ADU28225 | pyruvate ferredoxin/flavodoxin oxidoreductase | 1069 | 90.59% | 9566 |
| ADU28097 | glyceraldehyde-3-phosphate dehydrogenase, type I | 313 | 92.06% | 7526 |
| ADU26650 | glutamine synthetase catalytic region | 631 | 90.40% | 5006 |
| ADU26627 | translation elongation factor Tu | 348 | 87.00% | 4929 |
| ADU27083 | Phosphoglycerate kinase | 382 | 96.22% | 4570 |
| ADU26626 | translation elongation factor G | 605 | 87.18% | 4234 |
| ADU25642 | iron-containing alcohol dehydrogenase | 365 | 93.83% | 3750 |
| ADU28155 | enolase | 383 | 89.07% | 3711 |
